# Supplementary material for: Global diversity and antimicrobial resistance of typhoid fever pathogens: Insights from a meta-analysis of 13,000 Salmonella Typhi genomes
Source: eLife. 2023 Sep 12;12:e85867. doi: 10.7554/eLife.85867 (PMC10506625; doi:10.7554/eLife.85867)
Supplement: Supplementary file 4. [file elife-85867-supp4.zip › SuppFile4_country_summary.csv.html]

TyphoidGenomicsConsortiumWG1/genotypes/SuppTable4\_country\_summary.csv at main · typhoidgenomics/TyphoidGenomicsConsortiumWG1 · GitHub


Skip to content


Toggle navigation

Sign up

- Product

  - Actions

    Automate any workflow
  - Packages

    Host and manage packages
  - Security

    Find and fix vulnerabilities
  - Codespaces

    Instant dev environments
  - Copilot

    Write better code with AI
  - Code review

    Manage code changes
  - Issues

    Plan and track work
  - Discussions

    Collaborate outside of code

  Explore
  - All features
  - Documentation
  - GitHub Skills
  - Blog
- Solutions

  For
  - Enterprise
  - Teams
  - Startups
  - Education

  By Solution
  - CI/CD & Automation
  - DevOps
  - DevSecOps

  Case Studies
  - Customer Stories
  - Resources
- Open Source

  - GitHub Sponsors

    Fund open source developers

  - The ReadME Project

    GitHub community articles

  Repositories
  - Topics
  - Trending
  - Collections
- Pricing

- In this repository

  All GitHub
  ↵

  Jump to
  ↵

- No suggested jump to results

- In this repository

  All GitHub
  ↵

  Jump to
  ↵
- In this organization

  All GitHub
  ↵

  Jump to
  ↵
- In this repository

  All GitHub
  ↵

  Jump to
  ↵

Sign in

Sign up

You signed in with another tab or window. Reload to refresh your session.
You signed out in another tab or window. Reload to refresh your session.
You switched accounts on another tab or window. Reload to refresh your session.

{{ message }}

typhoidgenomics
 
/
**TyphoidGenomicsConsortiumWG1**
Public

- Notifications
- Fork
  2
- Star
   1

- Code
- Pull requests
  0
- Actions
- Security
- Insights

More


- Code
- Pull requests
- Actions
- Security
- Insights

Permalink

main

Switch branches/tags


Branches
Tags

Could not load branches


Nothing to show

{{ refName }}
default
View all branches

Could not load tags


Nothing to show


{{ refName }}
default
View all tags

# Name already in use

A tag already exists with the provided branch name. Many Git commands accept both tag and branch names, so creating this branch may cause unexpected behavior. Are you sure you want to create this branch?

 Cancel
 Create

## TyphoidGenomicsConsortiumWG1/genotypes/**SuppTable4\_country\_summary.csv**

 Go to file

 

- Go to file
  T
- Go to line
  L
- Copy path
- Copy permalink

This commit does not belong to any branch on this repository, and may belong to a fork outside of the repository.

Cannot retrieve contributors at this time

112 lines (112 sloc)
6.17 KB

Raw
 
Blame

Edit this file

E


Open in GitHub Desktop

- Open with Desktop
- View raw
- Copy raw contents
   Copy raw contents

   Copy raw contents

   Copy raw contents
- View blame

This file contains bidirectional Unicode text that may be interpreted or compiled differently than what appears below. To review, open the file in an editor that reveals hidden Unicode characters.
Learn more about bidirectional Unicode characters

Show hidden characters


|  | Region | Country\_Origin | Total genomes | Travel (%) | Representative cases from 2010 | Travel (%) amongst representative cases from 2010 |
| --- | --- | --- | --- | --- | --- | --- |
|  | Australia and New Zealand | Australia | 12 | 0 (0%) | 12 | 0 (0%) |
|  | Australia and New Zealand | New Zealand | 45 | 0 (0%) | 45 | 0 (0%) |
|  | Caribbean | Dominican Republic | 6 | 6 (100%) | 6 | 6 (100%) |
|  | Caribbean | Haiti | 12 | 12 (100%) | 12 | 12 (100%) |
|  | Caribbean | Jamaica | 1 | 1 (100%) | 1 | 1 (100%) |
|  | Caribbean | Turks and Caicos Islands | 1 | 1 (100%) | 1 | 1 (100%) |
|  | Central America | El Salvador | 19 | 19 (100%) | 19 | 19 (100%) |
|  | Central America | Guatemala | 22 | 22 (100%) | 22 | 22 (100%) |
|  | Central America | Mexico | 61 | 60 (98.4%) | 58 | 58 (100%) |
|  | Central America | Panama | 1 | 1 (100%) | 1 | 1 (100%) |
|  | Eastern Africa | Burundi | 2 | 2 (100%) | 2 | 2 (100%) |
|  | Eastern Africa | Comoros | 3 | 3 (100%) | 0 | 0 (NaN%) |
|  | Eastern Africa | Ethiopia | 4 | 2 (50%) | 3 | 1 (33.3%) |
|  | Eastern Africa | Kenya | 254 | 7 (2.8%) | 149 | 6 (4%) |
|  | Eastern Africa | Madagascar | 12 | 3 (25%) | 8 | 0 (0%) |
|  | Eastern Africa | Malawi | 569 | 0 (0%) | 558 | 0 (0%) |
|  | Eastern Africa | Mayotte | 3 | 0 (0%) | 3 | 0 (0%) |
|  | Eastern Africa | Mozambique | 1 | 1 (100%) | 1 | 1 (100%) |
|  | Eastern Africa | Rwanda | 52 | 1 (1.9%) | 27 | 1 (3.7%) |
|  | Eastern Africa | Tanzania | 35 | 9 (25.7%) | 18 | 7 (38.9%) |
|  | Eastern Africa | Uganda | 37 | 7 (18.9%) | 36 | 6 (16.7%) |
|  | Eastern Africa | Zambia | 24 | 2 (8.3%) | 0 | 0 (NaN%) |
|  | Eastern Africa | Zimbabwe | 110 | 25 (22.7%) | 25 | 25 (100%) |
|  | Eastern Asia | China | 5 | 5 (100%) | 3 | 3 (100%) |
|  | Eastern Asia | Japan | 4 | 1 (25%) | 0 | 0 (NaN%) |
|  | Eastern Asia | Mongolia | 1 | 1 (100%) | 0 | 0 (NaN%) |
|  | Eastern Asia | South Korea | 2 | 2 (100%) | 0 | 0 (NaN%) |
|  | Eastern Europe | Bulgaria | 1 | 1 (100%) | 1 | 1 (100%) |
|  | Eastern Europe | Russia | 2 | 1 (50%) | 0 | 0 (NaN%) |
|  | Melanesia | Fiji | 144 | 102 (70.8%) | 32 | 25 (78.1%) |
|  | Melanesia | Papua New Guinea | 86 | 45 (52.3%) | 5 | 5 (100%) |
|  | Melanesia | Vanuatu | 2 | 2 (100%) | 0 | 0 (NaN%) |
|  | Micronesia | Marshall Islands | 4 | 1 (25%) | 1 | 1 (100%) |
|  | Middle Africa | Angola | 5 | 5 (100%) | 2 | 2 (100%) |
|  | Middle Africa | Cameroon | 27 | 10 (37%) | 0 | 0 (NaN%) |
|  | Middle Africa | Central African Republic | 3 | 1 (33.3%) | 0 | 0 (NaN%) |
|  | Middle Africa | Chad | 2 | 2 (100%) | 2 | 2 (100%) |
|  | Middle Africa | Democratic Republic of Congo | 1 | 0 (0%) | 0 | 0 (NaN%) |
|  | Middle Africa | Democratic Republic of the Congo | 20 | 4 (20%) | 17 | 2 (11.8%) |
|  | Middle Africa | Gabon | 1 | 1 (100%) | 0 | 0 (NaN%) |
|  | Northern Africa | Algeria | 8 | 8 (100%) | 0 | 0 (NaN%) |
|  | Northern Africa | Egypt | 7 | 7 (100%) | 1 | 1 (100%) |
|  | Northern Africa | Morocco | 13 | 12 (92.3%) | 2 | 2 (100%) |
|  | Northern Africa | Sudan | 9 | 9 (100%) | 2 | 2 (100%) |
|  | Northern Africa | Tunisia | 4 | 3 (75%) | 1 | 1 (100%) |
|  | Northern America | USA | 167 | 2 (1.2%) | 140 | 2 (1.4%) |
|  | Northern Europe | Ireland | 1 | 1 (100%) | 0 | 0 (NaN%) |
|  | Northern Europe | United Kingdom | 108 | 0 (0%) | 105 | 0 (0%) |
|  | Polynesia | Samoa | 319 | 87 (27.3%) | 259 | 42 (16.2%) |
|  | Polynesia | Tonga | 5 | 5 (100%) | 3 | 3 (100%) |
|  | South America | Argentina | 11 | 0 (0%) | 0 | 0 (NaN%) |
|  | South America | Brazil | 1 | 1 (100%) | 1 | 1 (100%) |
|  | South America | Chile | 259 | 49 (18.9%) | 97 | 0 (0%) |
|  | South America | Colombia | 76 | 0 (0%) | 0 | 0 (NaN%) |
|  | South America | French Guiana | 5 | 2 (40%) | 3 | 0 (0%) |
|  | South America | Peru | 14 | 14 (100%) | 3 | 3 (100%) |
|  | South America | Suriname | 1 | 1 (100%) | 1 | 1 (100%) |
|  | South-eastern Asia | Cambodia | 279 | 6 (2.2%) | 221 | 5 (2.3%) |
|  | South-eastern Asia | Indonesia | 145 | 39 (26.9%) | 65 | 27 (41.5%) |
|  | South-eastern Asia | Laos | 139 | 2 (1.4%) | 27 | 1 (3.7%) |
|  | South-eastern Asia | Malaysia | 35 | 35 (100%) | 3 | 3 (100%) |
|  | South-eastern Asia | Myanmar | 52 | 13 (25%) | 49 | 10 (20.4%) |
|  | South-eastern Asia | Philippines | 209 | 19 (9.1%) | 206 | 16 (7.8%) |
|  | South-eastern Asia | Singapore | 5 | 2 (40%) | 4 | 1 (25%) |
|  | South-eastern Asia | Thailand | 51 | 7 (13.7%) | 5 | 5 (100%) |
|  | South-eastern Asia | Timor-Leste | 1 | 1 (100%) | 1 | 1 (100%) |
|  | South-eastern Asia | Viet Nam | 224 | 6 (2.7%) | 3 | 3 (100%) |
|  | Southern Africa | Namibia | 1 | 1 (100%) | 1 | 1 (100%) |
|  | Southern Africa | South Africa | 316 | 5 (1.6%) | 285 | 1 (0.4%) |
|  | Southern Asia | Afghanistan | 6 | 6 (100%) | 5 | 5 (100%) |
|  | Southern Asia | Bangladesh | 2268 | 264 (11.6%) | 1591 | 216 (13.6%) |
|  | Southern Asia | India | 2708 | 1241 (45.8%) | 2267 | 1038 (45.8%) |
|  | Southern Asia | Nepal | 1436 | 39 (2.7%) | 1275 | 21 (1.6%) |
|  | Southern Asia | Pakistan | 1810 | 783 (43.3%) | 1484 | 597 (40.2%) |
|  | Southern Asia | Sri Lanka | 6 | 6 (100%) | 1 | 1 (100%) |
|  | Southern Europe | Greece | 2 | 2 (100%) | 2 | 2 (100%) |
|  | Southern Europe | Italy | 1 | 1 (100%) | 1 | 1 (100%) |
|  | Southern Europe | Malta | 3 | 3 (100%) | 0 | 0 (NaN%) |
|  | Southern Europe | Portugal | 2 | 2 (100%) | 1 | 1 (100%) |
|  | Southern Europe | Spain | 2 | 2 (100%) | 2 | 2 (100%) |
|  | Western Africa | Benin | 4 | 4 (100%) | 0 | 0 (NaN%) |
|  | Western Africa | Burkina Faso | 17 | 3 (17.6%) | 14 | 0 (0%) |
|  | Western Africa | Cape Verde | 1 | 1 (100%) | 0 | 0 (NaN%) |
|  | Western Africa | Cote d'Ivoire | 4 | 4 (100%) | 0 | 0 (NaN%) |
|  | Western Africa | Gambia | 25 | 1 (4%) | 20 | 0 (0%) |
|  | Western Africa | Ghana | 109 | 8 (7.3%) | 68 | 5 (7.4%) |
|  | Western Africa | Guinea | 2 | 2 (100%) | 0 | 0 (NaN%) |
|  | Western Africa | Guinea-Bissau | 3 | 0 (0%) | 3 | 0 (0%) |
|  | Western Africa | Liberia | 1 | 1 (100%) | 0 | 0 (NaN%) |
|  | Western Africa | Mali | 5 | 5 (100%) | 1 | 1 (100%) |
|  | Western Africa | Mauritania | 2 | 2 (100%) | 0 | 0 (NaN%) |
|  | Western Africa | Niger | 4 | 4 (100%) | 1 | 1 (100%) |
|  | Western Africa | Nigeria | 192 | 42 (21.9%) | 150 | 25 (16.7%) |
|  | Western Africa | Senegal | 10 | 2 (20%) | 8 | 0 (0%) |
|  | Western Africa | Sierra Leone | 2 | 2 (100%) | 2 | 2 (100%) |
|  | Western Africa | Togo | 3 | 3 (100%) | 0 | 0 (NaN%) |
|  | Western Asia | Armenia | 1 | 1 (100%) | 0 | 0 (NaN%) |
|  | Western Asia | Iraq | 19 | 19 (100%) | 8 | 8 (100%) |
|  | Western Asia | Lebanon | 8 | 8 (100%) | 4 | 4 (100%) |
|  | Western Asia | Qatar | 1 | 1 (100%) | 1 | 1 (100%) |
|  | Western Asia | Saudi Arabia | 4 | 4 (100%) | 3 | 3 (100%) |
|  | Western Asia | State of Palestine | 1 | 1 (100%) | 0 | 0 (NaN%) |
|  | Western Asia | Syria | 4 | 4 (100%) | 1 | 1 (100%) |
|  | Western Asia | Turkey | 5 | 5 (100%) | 0 | 0 (NaN%) |
|  | Western Asia | United Arab Emirates | 4 | 4 (100%) | 4 | 4 (100%) |
|  | Western Europe | France | 4 | 0 (0%) | 0 | 0 (NaN%) |
|  | Western Europe | Germany | 1 | 1 (100%) | 1 | 1 (100%) |
|  | Western Europe | Netherlands | 1 | 1 (100%) | 1 | 1 (100%) |
|  | Western Europe | Switzerland | 1 | 1 (100%) | 1 | 1 (100%) |
|  | Unknown | Not Provided | 225 | 193 (85.8%) | 0 | 0 (NaN%) |
|  | Total | - | 12968 | 3381 (26.1%) | 9478 | 2284 (24.1%) |

 Go

## Footer

© 2023 GitHub, Inc.

### Footer navigation

- Terms
- Privacy
- Security
- Status
- Docs
- Contact GitHub
- Pricing
- API
- Training
- Blog
- About

You can’t perform that action at this time.
