# Supplementary material for: Global diversity and antimicrobial resistance of typhoid fever pathogens: Insights from a meta-analysis of 13,000 Salmonella Typhi genomes
Source: eLife. 2023 Sep 12;12:e85867. doi: 10.7554/eLife.85867 (PMC10506625; doi:10.7554/eLife.85867)
Supplement: Supplementary file 7. [file elife-85867-supp7.zip › SuppFile7_AMR_freqs_by_Region_20102020.csv.html]

TyphoidGenomicsConsortiumWG1/AMR/SuppTable7\_AMR\_freqs\_by\_Region\_2010-2020.csv at main · typhoidgenomics/TyphoidGenomicsConsortiumWG1 · GitHub


Skip to content


Toggle navigation

Sign up

- Product

  - Actions

    Automate any workflow
  - Packages

    Host and manage packages
  - Security

    Find and fix vulnerabilities
  - Codespaces

    Instant dev environments
  - Copilot

    Write better code with AI
  - Code review

    Manage code changes
  - Issues

    Plan and track work
  - Discussions

    Collaborate outside of code

  Explore
  - All features
  - Documentation
  - GitHub Skills
  - Blog
- Solutions

  For
  - Enterprise
  - Teams
  - Startups
  - Education

  By Solution
  - CI/CD & Automation
  - DevOps
  - DevSecOps

  Case Studies
  - Customer Stories
  - Resources
- Open Source

  - GitHub Sponsors

    Fund open source developers

  - The ReadME Project

    GitHub community articles

  Repositories
  - Topics
  - Trending
  - Collections
- Pricing

- In this repository

  All GitHub
  ↵

  Jump to
  ↵

- No suggested jump to results

- In this repository

  All GitHub
  ↵

  Jump to
  ↵
- In this organization

  All GitHub
  ↵

  Jump to
  ↵
- In this repository

  All GitHub
  ↵

  Jump to
  ↵

Sign in

Sign up

You signed in with another tab or window. Reload to refresh your session.
You signed out in another tab or window. Reload to refresh your session.
You switched accounts on another tab or window. Reload to refresh your session.

{{ message }}

typhoidgenomics
 
/
**TyphoidGenomicsConsortiumWG1**
Public

- Notifications
- Fork
  2
- Star
   1

- Code
- Pull requests
  0
- Actions
- Security
- Insights

More


- Code
- Pull requests
- Actions
- Security
- Insights

Permalink

main

Switch branches/tags


Branches
Tags

Could not load branches


Nothing to show

{{ refName }}
default
View all branches

Could not load tags


Nothing to show


{{ refName }}
default
View all tags

# Name already in use

A tag already exists with the provided branch name. Many Git commands accept both tag and branch names, so creating this branch may cause unexpected behavior. Are you sure you want to create this branch?

 Cancel
 Create

## TyphoidGenomicsConsortiumWG1/AMR/**SuppTable7\_AMR\_freqs\_by\_Region\_2010-2020.csv**

 Go to file

 

- Go to file
  T
- Go to line
  L
- Copy path
- Copy permalink

This commit does not belong to any branch on this repository, and may belong to a fork outside of the repository.

Cannot retrieve contributors at this time

22 lines (22 sloc)
1.48 KB

Raw
 
Blame

Edit this file

E


Open in GitHub Desktop

- Open with Desktop
- View raw
- Copy raw contents
   Copy raw contents

   Copy raw contents

   Copy raw contents
- View blame

This file contains bidirectional Unicode text that may be interpreted or compiled differently than what appears below. To review, open the file in an editor that reveals hidden Unicode characters.
Learn more about bidirectional Unicode characters

Show hidden characters


|  | Region | n | MDR | XDR | cipNS | cipR | cefR | aziR | MDR\_lower | MDR\_upper | XDR\_lower | XDR\_upper | cipNS\_lower | cipNS\_upper | cipR\_lower | cipR\_upper | cefR\_lower | cefR\_upper | aziR\_lower | aziR\_upper |
| --- | --- | --- | --- | --- | --- | --- | --- | --- | --- | --- | --- | --- | --- | --- | --- | --- | --- | --- | --- | --- |
|  | Australia and New Zealand | 57 | 1.8 | 0 | 19 | 0 | 0 | 0 | 0 | 5 | 0 | 0 | 9 | 30 | 0 | 0 | 0 | 0 | 0 | 0 |
|  | Caribbean | 20 | 0 | 0 | 5 | 0 | 0 | 0 | 0 | 0 | 0 | 0 | 0 | 15 | 0 | 0 | 0 | 0 | 0 | 0 |
|  | Central America | 100 | 2 | 1 | 38 | 2 | 1 | 0 | 0 | 5 | 0 | 3 | 28 | 48 | 0 | 5 | 0 | 3 | 0 | 0 |
|  | Eastern Africa | 830 | 83 | 0 | 21 | 0.12 | 0 | 0 | 80 | 85 | 0 | 0 | 18 | 24 | 0 | 0 | 0 | 0 | 0 | 0 |
|  | Eastern Asia | 3 | 0 | 0 | 67 | 33 | 0 | 0 | 0 | 0 | 0 | 0 | 13 | 100 | 0 | 87 | 0 | 0 | 0 | 0 |
|  | Eastern Europe | 1 | 0 | 0 | 100 | 0 | 0 | 0 | 0 | 0 | 0 | 0 | 100 | 100 | 0 | 0 | 0 | 0 | 0 | 0 |
|  | Melanesia | 37 | 0 | 0 | 0 | 0 | 0 | 0 | 0 | 0 | 0 | 0 | 0 | 0 | 0 | 0 | 0 | 0 | 0 | 0 |
|  | Micronesia | 1 | 0 | 0 | 0 | 0 | 0 | 0 | 0 | 0 | 0 | 0 | 0 | 0 | 0 | 0 | 0 | 0 | 0 | 0 |
|  | Middle Africa | 21 | 4.8 | 0 | 38 | 0 | 0 | 0 | 0 | 14 | 0 | 0 | 17 | 59 | 0 | 0 | 0 | 0 | 0 | 0 |
|  | Northern Africa | 6 | 0 | 0 | 50 | 0 | 0 | 0 | 0 | 0 | 0 | 0 | 10 | 90 | 0 | 0 | 0 | 0 | 0 | 0 |
|  | Northern America | 140 | 6.4 | 0.71 | 34 | 4.3 | 0.71 | 0 | 2 | 10 | 0 | 2 | 26 | 41 | 1 | 8 | 0 | 2 | 0 | 0 |
|  | Northern Europe | 105 | 21 | 0 | 68 | 6.7 | 0.95 | 0 | 13 | 29 | 0 | 0 | 59 | 77 | 2 | 11 | 0 | 3 | 0 | 0 |
|  | Polynesia | 262 | 0 | 0 | 5 | 0 | 0 | 0 | 0 | 0 | 0 | 0 | 2 | 8 | 0 | 0 | 0 | 0 | 0 | 0 |
|  | South America | 105 | 0 | 0 | 12 | 4.8 | 0 | 0 | 0 | 0 | 0 | 0 | 6 | 19 | 1 | 9 | 0 | 0 | 0 | 0 |
|  | South-eastern Asia | 584 | 29 | 0 | 52 | 3.3 | 0.17 | 0.34 | 25 | 33 | 0 | 0 | 48 | 56 | 2 | 5 | 0 | 1 | 0 | 1 |
|  | Southern Africa | 286 | 61 | 0 | 20 | 3.5 | 0 | 0 | 56 | 67 | 0 | 0 | 16 | 25 | 1 | 6 | 0 | 0 | 0 | 0 |
|  | Southern Asia | 6623 | 23 | 8.2 | 96 | 20 | 8.8 | 0.82 | 22 | 24 | 8 | 9 | 96 | 97 | 19 | 21 | 8 | 9 | 1 | 1 |
|  | Southern Europe | 6 | 0 | 0 | 50 | 17 | 0 | 0 | 0 | 0 | 0 | 0 | 10 | 90 | 0 | 46 | 0 | 0 | 0 | 0 |
|  | Western Africa | 267 | 39 | 0 | 12 | 0 | 0 | 0 | 33 | 44 | 0 | 0 | 8 | 16 | 0 | 0 | 0 | 0 | 0 | 0 |
|  | Western Asia | 21 | 29 | 0 | 76 | 9.5 | 9.5 | 0 | 9 | 48 | 0 | 0 | 58 | 94 | 0 | 22 | 0 | 22 | 0 | 0 |
|  | Western Europe | 3 | 0 | 0 | 33 | 0 | 0 | 0 | 0 | 0 | 0 | 0 | 0 | 87 | 0 | 0 | 0 | 0 | 0 | 0 |

 Go

## Footer

© 2023 GitHub, Inc.

### Footer navigation

- Terms
- Privacy
- Security
- Status
- Docs
- Contact GitHub
- Pricing
- API
- Training
- Blog
- About

You can’t perform that action at this time.
