# Supplementary material for: Global diversity and antimicrobial resistance of typhoid fever pathogens: Insights from a meta-analysis of 13,000 Salmonella Typhi genomes
Source: eLife. 2023 Sep 12;12:e85867. doi: 10.7554/eLife.85867 (PMC10506625; doi:10.7554/eLife.85867)
Supplement: Supplementary file 8. [file elife-85867-supp8.zip › SuppFile8_AMR_freqs_selectCountries_20002020.csv.html]

TyphoidGenomicsConsortiumWG1/AMR/SuppTable8\_AMR\_freqs\_selectCountries\_2000-2020.csv at main · typhoidgenomics/TyphoidGenomicsConsortiumWG1 · GitHub


Skip to content


Toggle navigation

Sign up

- Product

  - Actions

    Automate any workflow
  - Packages

    Host and manage packages
  - Security

    Find and fix vulnerabilities
  - Codespaces

    Instant dev environments
  - Copilot

    Write better code with AI
  - Code review

    Manage code changes
  - Issues

    Plan and track work
  - Discussions

    Collaborate outside of code

  Explore
  - All features
  - Documentation
  - GitHub Skills
  - Blog
- Solutions

  For
  - Enterprise
  - Teams
  - Startups
  - Education

  By Solution
  - CI/CD & Automation
  - DevOps
  - DevSecOps

  Case Studies
  - Customer Stories
  - Resources
- Open Source

  - GitHub Sponsors

    Fund open source developers

  - The ReadME Project

    GitHub community articles

  Repositories
  - Topics
  - Trending
  - Collections
- Pricing

- In this repository

  All GitHub
  ↵

  Jump to
  ↵

- No suggested jump to results

- In this repository

  All GitHub
  ↵

  Jump to
  ↵
- In this organization

  All GitHub
  ↵

  Jump to
  ↵
- In this repository

  All GitHub
  ↵

  Jump to
  ↵

Sign in

Sign up

You signed in with another tab or window. Reload to refresh your session.
You signed out in another tab or window. Reload to refresh your session.
You switched accounts on another tab or window. Reload to refresh your session.

{{ message }}

typhoidgenomics
 
/
**TyphoidGenomicsConsortiumWG1**
Public

- Notifications
- Fork
  2
- Star
   1

- Code
- Pull requests
  0
- Actions
- Security
- Insights

More


- Code
- Pull requests
- Actions
- Security
- Insights

Permalink

main

Switch branches/tags


Branches
Tags

Could not load branches


Nothing to show

{{ refName }}
default
View all branches

Could not load tags


Nothing to show


{{ refName }}
default
View all tags

# Name already in use

A tag already exists with the provided branch name. Many Git commands accept both tag and branch names, so creating this branch may cause unexpected behavior. Are you sure you want to create this branch?

 Cancel
 Create

## TyphoidGenomicsConsortiumWG1/AMR/**SuppTable8\_AMR\_freqs\_selectCountries\_2000-2020.csv**

 Go to file

 

- Go to file
  T
- Go to line
  L
- Copy path
- Copy permalink

This commit does not belong to any branch on this repository, and may belong to a fork outside of the repository.

Cannot retrieve contributors at this time

289 lines (289 sloc)
33.8 KB

Raw
 
Blame

Edit this file

E


Open in GitHub Desktop

- Open with Desktop
- View raw
- Copy raw contents
   Copy raw contents

   Copy raw contents

   Copy raw contents
- View blame

This file contains bidirectional Unicode text that may be interpreted or compiled differently than what appears below. To review, open the file in an editor that reveals hidden Unicode characters.
Learn more about bidirectional Unicode characters

Show hidden characters


|  | Country\_Origin | Year | n | sumMDR | MDR | sumXDR | XDR | sumcipNS | cipNS | sumcipR | cipR | sumcefR | cefR | sumaziR | aziR | sumMDR\_IncHI1 | IncHI1 amongst MDR | MDR\_lower | MDR\_upper | XDR\_lower | XDR\_upper | cipNS\_lower | cipNS\_upper | cipR\_lower | cipR\_upper | cefR\_lower | cefR\_upper | aziR\_lower | aziR\_upper |
| --- | --- | --- | --- | --- | --- | --- | --- | --- | --- | --- | --- | --- | --- | --- | --- | --- | --- | --- | --- | --- | --- | --- | --- | --- | --- | --- | --- | --- | --- |
|  | Afghanistan | all | 5 | 5 | 100 | 0 | 0 | 5 | 100 | 0 | 0 | 0 | 0 | 0 | 0 | NA | NA | 1 | 1 | 0 | 0 | 1 | 1 | 0 | 0 | 0 | 0 | 0 | 0 |
|  | Angola | all | 2 | 0 | 0 | 0 | 0 | 0 | 0 | 0 | 0 | 0 | 0 | 0 | 0 | NA | NA | 0 | 0 | 0 | 0 | 0 | 0 | 0 | 0 | 0 | 0 | 0 | 0 |
|  | Australia | all | 12 | 1 | 8.3 | 0 | 0 | 7 | 58 | 0 | 0 | 0 | 0 | 0 | 0 | NA | NA | -0.0733797609335352 | 0.239379760933535 | 0 | 0 | 0.301055229148878 | 0.858944770851122 | 0 | 0 | 0 | 0 | 0 | 0 |
|  | Bangladesh | 2000 | 1 | 0 | 0 | 0 | 0 | 0 | 0 | 0 | 0 | 0 | 0 | 0 | 0 | 0 | NA | 0 | 0 | 0 | 0 | 0 | 0 | 0 | 0 | 0 | 0 | 0 | 0 |
|  | Bangladesh | 2003 | 1 | 0 | 0 | 0 | 0 | 1 | 100 | 0 | 0 | 0 | 0 | 0 | 0 | 0 | NA | 0 | 0 | 0 | 0 | 1 | 1 | 0 | 0 | 0 | 0 | 0 | 0 |
|  | Bangladesh | 2004 | 3 | 2 | 67 | 0 | 0 | 2 | 67 | 0 | 0 | 0 | 0 | 0 | 0 | 0 | 0 | 0.136555567127219 | 1.20344443287278 | 0 | 0 | 0.136555567127219 | 1.20344443287278 | 0 | 0 | 0 | 0 | 0 | 0 |
|  | Bangladesh | 2005 | 11 | 10 | 91 | 0 | 0 | 11 | 100 | 0 | 0 | 0 | 0 | 0 | 0 | 2 | 20 | 0.740110302279876 | 1.07988969772012 | 0 | 0 | 1 | 1 | 0 | 0 | 0 | 0 | 0 | 0 |
|  | Bangladesh | 2006 | 3 | 2 | 67 | 0 | 0 | 2 | 67 | 0 | 0 | 0 | 0 | 0 | 0 | 0 | 0 | 0.136555567127219 | 1.20344443287278 | 0 | 0 | 0.136555567127219 | 1.20344443287278 | 0 | 0 | 0 | 0 | 0 | 0 |
|  | Bangladesh | 2007 | 13 | 5 | 38 | 0 | 0 | 12 | 92 | 1 | 8 | 0 | 0 | 0 | 0 | 0 | 0 | 0.115533274178188 | 0.644466725821812 | 0 | 0 | 0.77514560855786 | 1.06485439144214 | -0.0648543914421403 | 0.22485439144214 | 0 | 0 | 0 | 0 |
|  | Bangladesh | 2008 | 23 | 13 | 57 | 0 | 0 | 23 | 100 | 1 | 4 | 0 | 0 | 0 | 0 | 3 | 23 | 0.367401608284273 | 0.772598391715727 | 0 | 0 | 1 | 1 | -0.0433442528666764 | 0.123344252866676 | 0 | 0 | 0 | 0 |
|  | Bangladesh | 2009 | 17 | 10 | 59 | 0 | 0 | 17 | 100 | 3 | 18 | 0 | 0 | 0 | 0 | 0 | 0 | 0.356045335024953 | 0.823954664975047 | 0 | 0 | 1 | 1 | -0.00122050424317843 | 0.361220504243178 | 0 | 0 | 0 | 0 |
|  | Bangladesh | 2010 | 40 | 16 | 40 | 0 | 0 | 40 | 100 | 1 | 2 | 0 | 0 | 0 | 0 | 0 | 0 | 0.248179052828669 | 0.551820947171331 | 0 | 0 | 1 | 1 | -0.0283836232624222 | 0.0683836232624222 | 0 | 0 | 0 | 0 |
|  | Bangladesh | 2011 | 34 | 11 | 32 | 0 | 0 | 34 | 100 | 3 | 9 | 0 | 0 | 0 | 0 | 0 | 0 | 0.162747355963136 | 0.477252644036864 | 0 | 0 | 1 | 1 | -0.00534088305272912 | 0.185340883052729 | 0 | 0 | 0 | 0 |
|  | Bangladesh | 2012 | 69 | 11 | 16 | 0 | 0 | 68 | 99 | 3 | 4 | 0 | 0 | 0 | 0 | 0 | 0 | 0.0736240624017806 | 0.246375937598219 | 0 | 0 | 0.961800793255614 | 1.01819920674439 | -0.00811882682798385 | 0.0881188268279839 | 0 | 0 | 0 | 0 |
|  | Bangladesh | 2013 | 17 | 2 | 12 | 0 | 0 | 17 | 100 | 2 | 12 | 0 | 0 | 0 | 0 | 1 | 50 | -0.0331592802051694 | 0.273159280205169 | 0 | 0 | 1 | 1 | -0.0331592802051694 | 0.273159280205169 | 0 | 0 | 0 | 0 |
|  | Bangladesh | 2014 | 34 | 4 | 12 | 0 | 0 | 34 | 100 | 1 | 3 | 0 | 0 | 1 | 3 | 1 | 25 | 0.0117000343652742 | 0.228299965634726 | 0 | 0 | 1 | 1 | -0.0267929811071119 | 0.0867929811071119 | 0 | 0 | -0.0267929811071119 | 0.0867929811071119 |
|  | Bangladesh | 2015 | 32 | 5 | 16 | 0 | 0 | 32 | 100 | 0 | 0 | 0 | 0 | 0 | 0 | 0 | 0 | 0.0341949750730322 | 0.285805024926968 | 0 | 0 | 1 | 1 | 0 | 0 | 0 | 0 | 0 | 0 |
|  | Bangladesh | 2016 | 106 | 24 | 23 | 0 | 0 | 105 | 99 | 1 | 1 | 0 | 0 | 3 | 3 | 1 | 4 | 0.15032724763517 | 0.30967275236483 | 0 | 0 | 0.971596860296184 | 1.00840313970382 | -0.00840313970381613 | 0.0284031397038161 | 0 | 0 | -0.00157014039006383 | 0.0615701403900638 |
|  | Bangladesh | 2017 | 582 | 128 | 22 | 0 | 0 | 578 | 99 | 27 | 5 | 0 | 0 | 26 | 4 | 0 | 0 | 0.186348493943146 | 0.253651506056854 | 0 | 0 | 0.983287790405408 | 0.996712209594592 | 0.0329116563957406 | 0.0670883436042594 | 0 | 0 | 0.0232159925292805 | 0.0567840074707195 |
|  | Bangladesh | 2018 | 637 | 189 | 30 | 0 | 0 | 636 | 100 | 52 | 8 | 0 | 0 | 9 | 1 | 1 | 1 | 0.264525455157027 | 0.335474544842973 | 0 | 0 | 0.99692549303894 | 1.00307450696106 | 0.05873690264185 | 0.10126309735815 | 0 | 0 | 0.000834672258534695 | 0.0191653277414653 |
|  | Bangladesh | 2019 | 40 | 3 | 8 | 0 | 0 | 39 | 98 | 3 | 8 | 0 | 0 | 2 | 5 | 0 | 0 | -0.00162582311989265 | 0.161625823119893 | 0 | 0 | 0.931616376737578 | 1.02838362326242 | -0.00162582311989265 | 0.161625823119893 | 0 | 0 | -0.0175418388852421 | 0.117541838885242 |
|  | Bangladesh | all | 1591 | 393 | 25 | 0 | 0 | 1583 | 99 | 93 | 5.8 | 0 | 0 | 41 | 2.6 | NA | NA | 0.228807827220486 | 0.271192172779514 | 0 | 0 | 0.986524348266993 | 0.993475651733007 | 0.0464721535561349 | 0.069527846443865 | 0 | 0 | 0.0182141043407449 | 0.0337858956592551 |
|  | Brazil | all | 1 | 0 | 0 | 0 | 0 | 0 | 0 | 0 | 0 | 0 | 0 | 0 | 0 | NA | NA | 0 | 0 | 0 | 0 | 0 | 0 | 0 | 0 | 0 | 0 | 0 | 0 |
|  | Bulgaria | all | 1 | 0 | 0 | 0 | 0 | 1 | 100 | 0 | 0 | 0 | 0 | 0 | 0 | NA | NA | 0 | 0 | 0 | 0 | 1 | 1 | 0 | 0 | 0 | 0 | 0 | 0 |
|  | Burkina Faso | all | 14 | 0 | 0 | 0 | 0 | 0 | 0 | 0 | 0 | 0 | 0 | 0 | 0 | NA | NA | 0 | 0 | 0 | 0 | 0 | 0 | 0 | 0 | 0 | 0 | 0 | 0 |
|  | Burundi | all | 2 | 1 | 50 | 0 | 0 | 2 | 100 | 0 | 0 | 0 | 0 | 0 | 0 | NA | NA | -0.192964645562817 | 1.19296464556282 | 0 | 0 | 1 | 1 | 0 | 0 | 0 | 0 | 0 | 0 |
|  | Cambodia | 2007 | 16 | 12 | 75 | 0 | 0 | 13 | 81 | 0 | 0 | 0 | 0 | 0 | 0 | 12 | 100 | 0.537823776072813 | 0.962176223927187 | 0 | 0 | 0.618746936299049 | 1.00125306370095 | 0 | 0 | 0 | 0 | 0 | 0 |
|  | Cambodia | 2008 | 23 | 17 | 74 | 0 | 0 | 23 | 100 | 0 | 0 | 0 | 0 | 0 | 0 | 17 | 100 | 0.560541347182668 | 0.919458652817332 | 0 | 0 | 1 | 1 | 0 | 0 | 0 | 0 | 0 | 0 |
|  | Cambodia | 2009 | 17 | 12 | 71 | 0 | 0 | 16 | 94 | 0 | 0 | 0 | 0 | 0 | 0 | 12 | 100 | 0.493400068730548 | 0.926599931269452 | 0 | 0 | 0.828148276453832 | 1.05185172354617 | 0 | 0 | 0 | 0 | 0 | 0 |
|  | Cambodia | 2010 | 43 | 33 | 77 | 0 | 0 | 41 | 95 | 0 | 0 | 0 | 0 | 0 | 0 | 33 | 100 | 0.643727040895572 | 0.896272959104428 | 0 | 0 | 0.887055133877696 | 1.0129448661223 | 0 | 0 | 0 | 0 | 0 | 0 |
|  | Cambodia | 2011 | 56 | 48 | 86 | 0 | 0 | 55 | 98 | 0 | 0 | 0 | 0 | 0 | 0 | 48 | 100 | 0.768348486100883 | 0.951651513899117 | 0 | 0 | 0.945313907686221 | 1.01468609231378 | 0 | 0 | 0 | 0 | 0 | 0 |
|  | Cambodia | 2012 | 90 | 79 | 88 | 0 | 0 | 89 | 99 | 0 | 0 | 0 | 0 | 0 | 0 | 79 | 100 | 0.812329062147005 | 0.947670937852995 | 0 | 0 | 0.968343547833522 | 1.01165645216648 | 0 | 0 | 0 | 0 | 0 | 0 |
|  | Cambodia | 2013 | 15 | 2 | 13 | 0 | 0 | 13 | 87 | 0 | 0 | 0 | 0 | 0 | 0 | 2 | 100 | -0.0420306604886122 | 0.302030660488612 | 0 | 0 | 0.697969339511388 | 1.04203066048861 | 0 | 0 | 0 | 0 | 0 | 0 |
|  | Cambodia | 2014 | 6 | 1 | 17 | 0 | 0 | 6 | 100 | 0 | 0 | 0 | 0 | 0 | 0 | 1 | 100 | -0.12820450353059 | 0.46820450353059 | 0 | 0 | 1 | 1 | 0 | 0 | 0 | 0 | 0 | 0 |
|  | Cambodia | 2015 | 7 | 2 | 29 | 0 | 0 | 6 | 86 | 0 | 0 | 0 | 0 | 0 | 0 | 2 | 100 | -0.0446640106136302 | 0.62466401061363 | 0 | 0 | 0.600770372063686 | 1.11922962793631 | 0 | 0 | 0 | 0 | 0 | 0 |
|  | Cambodia | 2018 | 2 | 2 | 100 | 0 | 0 | 2 | 100 | 0 | 0 | 0 | 0 | 0 | 0 | 2 | 100 | 1 | 1 | 0 | 0 | 1 | 1 | 0 | 0 | 0 | 0 | 0 | 0 |
|  | Cambodia | 2019 | 2 | 0 | 0 | 0 | 0 | 1 | 50 | 0 | 0 | 0 | 0 | 0 | 0 | 0 | NA | 0 | 0 | 0 | 0 | -0.192964645562817 | 1.19296464556282 | 0 | 0 | 0 | 0 | 0 | 0 |
|  | Cambodia | all | 221 | 167 | 76 | 0 | 0 | 213 | 96 | 0 | 0 | 0 | 0 | 0 | 0 | NA | NA | 0.70334701775422 | 0.81665298224578 | 0 | 0 | 0.93537351342799 | 0.98462648657201 | 0 | 0 | 0 | 0 | 0 | 0 |
|  | Chad | all | 2 | 0 | 0 | 0 | 0 | 1 | 50 | 0 | 0 | 0 | 0 | 0 | 0 | NA | NA | 0 | 0 | 0 | 0 | -0.192964645562817 | 1.19296464556282 | 0 | 0 | 0 | 0 | 0 | 0 |
|  | Chile | 2011 | 8 | 0 | 0 | 0 | 0 | 0 | 0 | 0 | 0 | 0 | 0 | 0 | 0 | 0 | NA | 0 | 0 | 0 | 0 | 0 | 0 | 0 | 0 | 0 | 0 | 0 | 0 |
|  | Chile | 2012 | 35 | 0 | 0 | 0 | 0 | 1 | 3 | 1 | 3 | 0 | 0 | 0 | 0 | 0 | NA | 0 | 0 | 0 | 0 | -0.0251942025941131 | 0.0851942025941131 | -0.0251942025941131 | 0.0851942025941131 | 0 | 0 | 0 | 0 |
|  | Chile | 2013 | 10 | 0 | 0 | 0 | 0 | 0 | 0 | 0 | 0 | 0 | 0 | 0 | 0 | 0 | NA | 0 | 0 | 0 | 0 | 0 | 0 | 0 | 0 | 0 | 0 | 0 | 0 |
|  | Chile | 2014 | 8 | 0 | 0 | 0 | 0 | 0 | 0 | 0 | 0 | 0 | 0 | 0 | 0 | 0 | NA | 0 | 0 | 0 | 0 | 0 | 0 | 0 | 0 | 0 | 0 | 0 | 0 |
|  | Chile | 2015 | 14 | 0 | 0 | 0 | 0 | 9 | 64 | 4 | 29 | 0 | 0 | 0 | 0 | 0 | NA | 0 | 0 | 0 | 0 | 0.389001992039777 | 0.890998007960223 | 0.0533568086760153 | 0.526643191323985 | 0 | 0 | 0 | 0 |
|  | Chile | 2016 | 8 | 0 | 0 | 0 | 0 | 1 | 12 | 0 | 0 | 0 | 0 | 0 | 0 | 0 | NA | 0 | 0 | 0 | 0 | -0.109176514939904 | 0.349176514939904 | 0 | 0 | 0 | 0 | 0 | 0 |
|  | Chile | 2017 | 7 | 0 | 0 | 0 | 0 | 0 | 0 | 0 | 0 | 0 | 0 | 0 | 0 | 0 | NA | 0 | 0 | 0 | 0 | 0 | 0 | 0 | 0 | 0 | 0 | 0 | 0 |
|  | Chile | 2018 | 7 | 0 | 0 | 0 | 0 | 0 | 0 | 0 | 0 | 0 | 0 | 0 | 0 | 0 | NA | 0 | 0 | 0 | 0 | 0 | 0 | 0 | 0 | 0 | 0 | 0 | 0 |
|  | Chile | all | 97 | 0 | 0 | 0 | 0 | 11 | 11 | 5 | 5.2 | 0 | 0 | 0 | 0 | NA | NA | 0 | 0 | 0 | 0 | 0.0468978672187716 | 0.173102132781228 | 0.00799749653589991 | 0.0960025034641001 | 0 | 0 | 0 | 0 |
|  | China | all | 3 | 0 | 0 | 0 | 0 | 2 | 67 | 1 | 33 | 0 | 0 | 0 | 0 | NA | NA | 0 | 0 | 0 | 0 | 0.136555567127219 | 1.20344443287278 | -0.203444432872781 | 0.863444432872781 | 0 | 0 | 0 | 0 |
|  | Democratic Republic of the Congo | all | 17 | 1 | 5.9 | 0 | 0 | 7 | 41 | 0 | 0 | 0 | 0 | 0 | 0 | NA | NA | -0.0528517235461677 | 0.170851723546168 | 0 | 0 | 0.176045335024953 | 0.643954664975047 | 0 | 0 | 0 | 0 | 0 | 0 |
|  | Dominican Republic | all | 6 | 0 | 0 | 0 | 0 | 0 | 0 | 0 | 0 | 0 | 0 | 0 | 0 | NA | NA | 0 | 0 | 0 | 0 | 0 | 0 | 0 | 0 | 0 | 0 | 0 | 0 |
|  | Egypt | all | 1 | 0 | 0 | 0 | 0 | 1 | 100 | 0 | 0 | 0 | 0 | 0 | 0 | NA | NA | 0 | 0 | 0 | 0 | 1 | 1 | 0 | 0 | 0 | 0 | 0 | 0 |
|  | El Salvador | all | 19 | 0 | 0 | 0 | 0 | 15 | 79 | 0 | 0 | 0 | 0 | 0 | 0 | NA | NA | 0 | 0 | 0 | 0 | 0.606683610461923 | 0.973316389538077 | 0 | 0 | 0 | 0 | 0 | 0 |
|  | Ethiopia | all | 3 | 0 | 0 | 0 | 0 | 0 | 0 | 0 | 0 | 0 | 0 | 0 | 0 | NA | NA | 0 | 0 | 0 | 0 | 0 | 0 | 0 | 0 | 0 | 0 | 0 | 0 |
|  | Fiji | all | 32 | 0 | 0 | 0 | 0 | 0 | 0 | 0 | 0 | 0 | 0 | 0 | 0 | NA | NA | 0 | 0 | 0 | 0 | 0 | 0 | 0 | 0 | 0 | 0 | 0 | 0 |
|  | French Guiana | all | 3 | 0 | 0 | 0 | 0 | 0 | 0 | 0 | 0 | 0 | 0 | 0 | 0 | NA | NA | 0 | 0 | 0 | 0 | 0 | 0 | 0 | 0 | 0 | 0 | 0 | 0 |
|  | Gambia | all | 20 | 0 | 0 | 0 | 0 | 0 | 0 | 0 | 0 | 0 | 0 | 0 | 0 | NA | NA | 0 | 0 | 0 | 0 | 0 | 0 | 0 | 0 | 0 | 0 | 0 | 0 |
|  | Germany | all | 1 | 0 | 0 | 0 | 0 | 0 | 0 | 0 | 0 | 0 | 0 | 0 | 0 | NA | NA | 0 | 0 | 0 | 0 | 0 | 0 | 0 | 0 | 0 | 0 | 0 | 0 |
|  | Ghana | all | 68 | 45 | 66 | 0 | 0 | 0 | 0 | 0 | 0 | 0 | 0 | 0 | 0 | NA | NA | 0.547549115138172 | 0.772450884861828 | 0 | 0 | 0 | 0 | 0 | 0 | 0 | 0 | 0 | 0 |
|  | Greece | all | 2 | 0 | 0 | 0 | 0 | 2 | 100 | 1 | 50 | 0 | 0 | 0 | 0 | NA | NA | 0 | 0 | 0 | 0 | 1 | 1 | -0.192964645562817 | 1.19296464556282 | 0 | 0 | 0 | 0 |
|  | Guatemala | all | 22 | 0 | 0 | 0 | 0 | 8 | 36 | 0 | 0 | 0 | 0 | 0 | 0 | NA | NA | 0 | 0 | 0 | 0 | 0.158983798792214 | 0.561016201207786 | 0 | 0 | 0 | 0 | 0 | 0 |
|  | Guinea-Bissau | all | 3 | 0 | 0 | 0 | 0 | 0 | 0 | 0 | 0 | 0 | 0 | 0 | 0 | NA | NA | 0 | 0 | 0 | 0 | 0 | 0 | 0 | 0 | 0 | 0 | 0 | 0 |
|  | Haiti | all | 12 | 0 | 0 | 0 | 0 | 0 | 0 | 0 | 0 | 0 | 0 | 0 | 0 | NA | NA | 0 | 0 | 0 | 0 | 0 | 0 | 0 | 0 | 0 | 0 | 0 | 0 |
|  | India | 2000 | 2 | 0 | 0 | 0 | 0 | 1 | 50 | 0 | 0 | 0 | 0 | 0 | 0 | 0 | NA | 0 | 0 | 0 | 0 | -0.192964645562817 | 1.19296464556282 | 0 | 0 | 0 | 0 | 0 | 0 |
|  | India | 2002 | 5 | 0 | 0 | 0 | 0 | 2 | 40 | 0 | 0 | 0 | 0 | 0 | 0 | 0 | NA | 0 | 0 | 0 | 0 | -0.0294144850840502 | 0.82941448508405 | 0 | 0 | 0 | 0 | 0 | 0 |
|  | India | 2003 | 1 | 0 | 0 | 0 | 0 | 1 | 100 | 0 | 0 | 0 | 0 | 0 | 0 | 0 | NA | 0 | 0 | 0 | 0 | 1 | 1 | 0 | 0 | 0 | 0 | 0 | 0 |
|  | India | 2004 | 5 | 0 | 0 | 0 | 0 | 4 | 80 | 0 | 0 | 0 | 0 | 0 | 0 | 0 | NA | 0 | 0 | 0 | 0 | 0.449384541128033 | 1.15061545887197 | 0 | 0 | 0 | 0 | 0 | 0 |
|  | India | 2005 | 6 | 3 | 50 | 0 | 0 | 5 | 83 | 0 | 0 | 0 | 0 | 0 | 0 | 1 | 33 | 0.0999166753454143 | 0.900083324654586 | 0 | 0 | 0.53179549646941 | 1.12820450353059 | 0 | 0 | 0 | 0 | 0 | 0 |
|  | India | 2006 | 7 | 1 | 14 | 0 | 0 | 5 | 71 | 0 | 0 | 0 | 0 | 0 | 0 | 1 | 100 | -0.119229627936314 | 0.399229627936314 | 0 | 0 | 0.37533598938637 | 1.04466401061363 | 0 | 0 | 0 | 0 | 0 | 0 |
|  | India | 2007 | 5 | 0 | 0 | 0 | 0 | 5 | 100 | 0 | 0 | 0 | 0 | 0 | 0 | 0 | NA | 0 | 0 | 0 | 0 | 1 | 1 | 0 | 0 | 0 | 0 | 0 | 0 |
|  | India | 2008 | 7 | 0 | 0 | 0 | 0 | 7 | 100 | 1 | 14 | 0 | 0 | 0 | 0 | 0 | NA | 0 | 0 | 0 | 0 | 1 | 1 | -0.119229627936314 | 0.399229627936314 | 0 | 0 | 0 | 0 |
|  | India | 2009 | 15 | 2 | 13 | 0 | 0 | 15 | 100 | 1 | 7 | 0 | 0 | 0 | 0 | 0 | 0 | -0.0420306604886122 | 0.302030660488612 | 0 | 0 | 1 | 1 | -0.056235992989028 | 0.196235992989028 | 0 | 0 | 0 | 0 |
|  | India | 2010 | 21 | 2 | 10 | 0 | 0 | 20 | 95 | 3 | 14 | 0 | 0 | 0 | 0 | 2 | 100 | -0.0255506390384492 | 0.225550639038449 | 0 | 0 | 0.85891599319147 | 1.04108400680853 | -0.00966629547095763 | 0.289666295470958 | 0 | 0 | 0 | 0 |
|  | India | 2011 | 93 | 13 | 14 | 0 | 0 | 91 | 98 | 13 | 14 | 0 | 0 | 0 | 0 | 0 | 0 | 0.0695228524839342 | 0.210477147516066 | 0 | 0 | 0.950517293944581 | 1.00948270605542 | 0.0695228524839342 | 0.210477147516066 | 0 | 0 | 0 | 0 |
|  | India | 2012 | 23 | 4 | 17 | 0 | 0 | 21 | 91 | 3 | 13 | 0 | 0 | 0 | 0 | 0 | 0 | 0.0150929749628769 | 0.324907025037123 | 0 | 0 | 0.794843365878551 | 1.02515663412145 | -0.00763850365660926 | 0.267638503656609 | 0 | 0 | 0 | 0 |
|  | India | 2014 | 49 | 0 | 0 | 0 | 0 | 47 | 96 | 13 | 27 | 0 | 0 | 1 | 2 | 0 | NA | 0 | 0 | 0 | 0 | 0.90459794448667 | 1.01540205551333 | 0.146381099126949 | 0.393618900873051 | 0 | 0 | -0.0195897327444315 | 0.0595897327444315 |
|  | India | 2015 | 64 | 5 | 8 | 0 | 0 | 63 | 98 | 12 | 19 | 0 | 0 | 0 | 0 | 0 | 0 | 0.0142497939196433 | 0.145750206080357 | 0 | 0 | 0.949615199786993 | 1.01038480021301 | 0.0943734681495245 | 0.285626531850475 | 0 | 0 | 0 | 0 |
|  | India | 2016 | 240 | 5 | 2 | 0 | 0 | 235 | 98 | 45 | 19 | 0 | 0 | 0 | 0 | 0 | 0 | 0.001930000435549 | 0.038069999564451 | 0 | 0 | 0.961930000435549 | 0.998069999564451 | 0.140618671291671 | 0.239381328708329 | 0 | 0 | 0 | 0 |
|  | India | 2017 | 472 | 7 | 1 | 0 | 0 | 470 | 100 | 115 | 24 | 1 | 0 | 6 | 1 | 0 | 0 | -0.000904821721217166 | 0.0209048217212172 | 0 | 0 | 0.9941398733764 | 1.0058601266236 | 0.201271907971308 | 0.278728092028692 | -0.0041481411608626 | 0.0041481411608626 | -0.000106753113571048 | 0.020106753113571 |
|  | India | 2018 | 797 | 15 | 2 | 0 | 0 | 787 | 99 | 196 | 25 | 12 | 2 | 3 | 0 | 0 | 0 | 0.0105655280995091 | 0.0294344719004909 | 0 | 0 | 0.982272198546502 | 0.997727801453497 | 0.220102569778852 | 0.279897430221148 | 0.011545381011983 | 0.028454618988017 | -0.00425147341610474 | 0.00425147341610474 |
|  | India | 2019 | 470 | 16 | 3 | 1 | 0 | 465 | 99 | 88 | 19 | 1 | 0 | 1 | 0 | 0 | 0 | 0.0136055367633105 | 0.0463944632366895 | -0.00416577400709876 | 0.00416577400709876 | 0.980724853818489 | 0.999275146181511 | 0.154731880635745 | 0.225268119364255 | -0.00416577400709876 | 0.00416577400709876 | -0.00416577400709876 | 0.00416577400709876 |
|  | India | 2020 | 36 | 0 | 0 | 0 | 0 | 36 | 100 | 8 | 22 | 1 | 3 | 0 | 0 | 0 | NA | 0 | 0 | 0 | 0 | 1 | 1 | 0.0841916948504273 | 0.355808305149573 | -0.023682946179978 | 0.083682946179978 | 0 | 0 |
|  | India | 2021 | 2 | 0 | 0 | 0 | 0 | 2 | 100 | 0 | 0 | 0 | 0 | 0 | 0 | 0 | NA | 0 | 0 | 0 | 0 | 1 | 1 | 0 | 0 | 0 | 0 | 0 | 0 |
|  | India | all | 2267 | 67 | 3 | 1 | 0.044 | 2237 | 99 | 496 | 22 | 15 | 0.66 | 11 | 0.49 | NA | NA | 0.0230284791121067 | 0.0369715208878933 | -0.000424388029531676 | 0.00130438802953168 | 0.985295944786744 | 0.994704055213256 | 0.202981200964193 | 0.237018799035807 | 0.00326259729365619 | 0.00993740270634381 | 0.00203948202328864 | 0.00776051797671136 |
|  | Indonesia | 2000 | 1 | 0 | 0 | 0 | 0 | 0 | 0 | 0 | 0 | 0 | 0 | 0 | 0 | 0 | NA | 0 | 0 | 0 | 0 | 0 | 0 | 0 | 0 | 0 | 0 | 0 | 0 |
|  | Indonesia | 2002 | 2 | 0 | 0 | 0 | 0 | 0 | 0 | 0 | 0 | 0 | 0 | 0 | 0 | 0 | NA | 0 | 0 | 0 | 0 | 0 | 0 | 0 | 0 | 0 | 0 | 0 | 0 |
|  | Indonesia | 2003 | 2 | 0 | 0 | 0 | 0 | 0 | 0 | 0 | 0 | 0 | 0 | 0 | 0 | 0 | NA | 0 | 0 | 0 | 0 | 0 | 0 | 0 | 0 | 0 | 0 | 0 | 0 |
|  | Indonesia | 2004 | 3 | 0 | 0 | 0 | 0 | 0 | 0 | 0 | 0 | 0 | 0 | 0 | 0 | 0 | NA | 0 | 0 | 0 | 0 | 0 | 0 | 0 | 0 | 0 | 0 | 0 | 0 |
|  | Indonesia | 2005 | 4 | 0 | 0 | 0 | 0 | 0 | 0 | 0 | 0 | 0 | 0 | 0 | 0 | 0 | NA | 0 | 0 | 0 | 0 | 0 | 0 | 0 | 0 | 0 | 0 | 0 | 0 |
|  | Indonesia | 2006 | 7 | 0 | 0 | 0 | 0 | 0 | 0 | 0 | 0 | 0 | 0 | 0 | 0 | 0 | NA | 0 | 0 | 0 | 0 | 0 | 0 | 0 | 0 | 0 | 0 | 0 | 0 |
|  | Indonesia | 2007 | 5 | 0 | 0 | 0 | 0 | 0 | 0 | 0 | 0 | 0 | 0 | 0 | 0 | 0 | NA | 0 | 0 | 0 | 0 | 0 | 0 | 0 | 0 | 0 | 0 | 0 | 0 |
|  | Indonesia | 2008 | 11 | 0 | 0 | 0 | 0 | 0 | 0 | 0 | 0 | 0 | 0 | 0 | 0 | 0 | NA | 0 | 0 | 0 | 0 | 0 | 0 | 0 | 0 | 0 | 0 | 0 | 0 |
|  | Indonesia | 2009 | 32 | 0 | 0 | 0 | 0 | 0 | 0 | 0 | 0 | 0 | 0 | 0 | 0 | 0 | NA | 0 | 0 | 0 | 0 | 0 | 0 | 0 | 0 | 0 | 0 | 0 | 0 |
|  | Indonesia | 2010 | 31 | 0 | 0 | 0 | 0 | 1 | 3 | 0 | 0 | 0 | 0 | 0 | 0 | 0 | NA | 0 | 0 | 0 | 0 | -0.0321976760388652 | 0.0921976760388652 | 0 | 0 | 0 | 0 | 0 | 0 |
|  | Indonesia | 2011 | 17 | 0 | 0 | 0 | 0 | 2 | 12 | 0 | 0 | 0 | 0 | 0 | 0 | 0 | NA | 0 | 0 | 0 | 0 | -0.0331592802051694 | 0.273159280205169 | 0 | 0 | 0 | 0 | 0 | 0 |
|  | Indonesia | 2012 | 5 | 0 | 0 | 0 | 0 | 0 | 0 | 0 | 0 | 0 | 0 | 0 | 0 | 0 | NA | 0 | 0 | 0 | 0 | 0 | 0 | 0 | 0 | 0 | 0 | 0 | 0 |
|  | Indonesia | 2014 | 2 | 0 | 0 | 0 | 0 | 0 | 0 | 0 | 0 | 0 | 0 | 0 | 0 | 0 | NA | 0 | 0 | 0 | 0 | 0 | 0 | 0 | 0 | 0 | 0 | 0 | 0 |
|  | Indonesia | 2015 | 1 | 0 | 0 | 0 | 0 | 0 | 0 | 0 | 0 | 0 | 0 | 0 | 0 | 0 | NA | 0 | 0 | 0 | 0 | 0 | 0 | 0 | 0 | 0 | 0 | 0 | 0 |
|  | Indonesia | 2016 | 1 | 0 | 0 | 0 | 0 | 0 | 0 | 0 | 0 | 0 | 0 | 0 | 0 | 0 | NA | 0 | 0 | 0 | 0 | 0 | 0 | 0 | 0 | 0 | 0 | 0 | 0 |
|  | Indonesia | 2017 | 4 | 0 | 0 | 0 | 0 | 0 | 0 | 0 | 0 | 0 | 0 | 0 | 0 | 0 | NA | 0 | 0 | 0 | 0 | 0 | 0 | 0 | 0 | 0 | 0 | 0 | 0 |
|  | Indonesia | 2018 | 2 | 0 | 0 | 0 | 0 | 0 | 0 | 0 | 0 | 0 | 0 | 0 | 0 | 0 | NA | 0 | 0 | 0 | 0 | 0 | 0 | 0 | 0 | 0 | 0 | 0 | 0 |
|  | Indonesia | 2019 | 2 | 0 | 0 | 0 | 0 | 0 | 0 | 0 | 0 | 0 | 0 | 0 | 0 | 0 | NA | 0 | 0 | 0 | 0 | 0 | 0 | 0 | 0 | 0 | 0 | 0 | 0 |
|  | Indonesia | all | 65 | 0 | 0 | 0 | 0 | 3 | 4.6 | 0 | 0 | 0 | 0 | 0 | 0 | NA | NA | 0 | 0 | 0 | 0 | -0.00500849483457663 | 0.0970084948345766 | 0 | 0 | 0 | 0 | 0 | 0 |
|  | Iraq | all | 8 | 2 | 25 | 0 | 0 | 5 | 62 | 0 | 0 | 2 | 25 | 0 | 0 | NA | NA | -0.0500624934909393 | 0.550062493490939 | 0 | 0 | 0.284519933528086 | 0.955480066471914 | 0 | 0 | -0.0500624934909393 | 0.550062493490939 | 0 | 0 |
|  | Italy | all | 1 | 0 | 0 | 0 | 0 | 1 | 100 | 0 | 0 | 0 | 0 | 0 | 0 | NA | NA | 0 | 0 | 0 | 0 | 1 | 1 | 0 | 0 | 0 | 0 | 0 | 0 |
|  | Jamaica | all | 1 | 0 | 0 | 0 | 0 | 1 | 100 | 0 | 0 | 0 | 0 | 0 | 0 | NA | NA | 0 | 0 | 0 | 0 | 1 | 1 | 0 | 0 | 0 | 0 | 0 | 0 |
|  | Kenya | 2001 | 1 | 1 | 100 | 0 | 0 | 1 | 100 | 0 | 0 | 0 | 0 | 0 | 0 | 1 | 100 | 1 | 1 | 0 | 0 | 1 | 1 | 0 | 0 | 0 | 0 | 0 | 0 |
|  | Kenya | 2004 | 1 | 1 | 100 | 0 | 0 | 0 | 0 | 0 | 0 | 0 | 0 | 0 | 0 | 1 | 100 | 1 | 1 | 0 | 0 | 0 | 0 | 0 | 0 | 0 | 0 | 0 | 0 |
|  | Kenya | 2007 | 16 | 11 | 69 | 0 | 0 | 7 | 44 | 0 | 0 | 0 | 0 | 0 | 0 | 11 | 100 | 0.462878921332695 | 0.917121078667305 | 0 | 0 | 0.196921598295941 | 0.683078401704059 | 0 | 0 | 0 | 0 | 0 | 0 |
|  | Kenya | 2008 | 33 | 26 | 79 | 0 | 0 | 2 | 6 | 0 | 0 | 0 | 0 | 0 | 0 | 26 | 100 | 0.650517136201303 | 0.929482863798697 | 0 | 0 | -0.0214106095158176 | 0.141410609515818 | 0 | 0 | 0 | 0 | 0 | 0 |
|  | Kenya | 2009 | 4 | 2 | 50 | 0 | 0 | 0 | 0 | 0 | 0 | 0 | 0 | 0 | 0 | 2 | 100 | 0.01 | 0.99 | 0 | 0 | 0 | 0 | 0 | 0 | 0 | 0 | 0 | 0 |
|  | Kenya | 2012 | 50 | 41 | 82 | 0 | 0 | 10 | 20 | 0 | 0 | 0 | 0 | 0 | 0 | 41 | 100 | 0.713508670775504 | 0.926491329224496 | 0 | 0 | 0.0891256567099493 | 0.310874343290051 | 0 | 0 | 0 | 0 | 0 | 0 |
|  | Kenya | 2013 | 14 | 13 | 93 | 0 | 0 | 9 | 64 | 0 | 0 | 0 | 0 | 0 | 0 | 13 | 100 | 0.79509262436768 | 1.06490737563232 | 0 | 0 | 0.389001992039777 | 0.890998007960223 | 0 | 0 | 0 | 0 | 0 | 0 |
|  | Kenya | 2014 | 13 | 9 | 69 | 0 | 0 | 7 | 54 | 0 | 0 | 0 | 0 | 0 | 0 | 7 | 78 | 0.439104834322742 | 0.940895165677257 | 0 | 0 | 0.269002248126936 | 0.810997751873064 | 0 | 0 | 0 | 0 | 0 | 0 |
|  | Kenya | 2015 | 34 | 30 | 88 | 0 | 0 | 26 | 76 | 0 | 0 | 0 | 0 | 0 | 0 | 19 | 63 | 0.771700034365274 | 0.988299965634726 | 0 | 0 | 0.617416469752276 | 0.902583530247724 | 0 | 0 | 0 | 0 | 0 | 0 |
|  | Kenya | 2016 | 34 | 19 | 56 | 0 | 0 | 22 | 65 | 0 | 0 | 0 | 0 | 0 | 0 | 7 | 37 | 0.393098543830059 | 0.726901456169941 | 0 | 0 | 0.489365191741447 | 0.810634808258553 | 0 | 0 | 0 | 0 | 0 | 0 |
|  | Kenya | 2017 | 2 | 2 | 100 | 0 | 0 | 2 | 100 | 0 | 0 | 0 | 0 | 0 | 0 | 0 | 0 | 1 | 1 | 0 | 0 | 1 | 1 | 0 | 0 | 0 | 0 | 0 | 0 |
|  | Kenya | 2018 | 1 | 1 | 100 | 0 | 0 | 1 | 100 | 0 | 0 | 0 | 0 | 0 | 0 | 0 | 0 | 1 | 1 | 0 | 0 | 1 | 1 | 0 | 0 | 0 | 0 | 0 | 0 |
|  | Kenya | 2019 | 1 | 1 | 100 | 0 | 0 | 1 | 100 | 0 | 0 | 0 | 0 | 0 | 0 | 0 | 0 | 1 | 1 | 0 | 0 | 1 | 1 | 0 | 0 | 0 | 0 | 0 | 0 |
|  | Kenya | all | 149 | 116 | 78 | 0 | 0 | 78 | 52 | 0 | 0 | 0 | 0 | 0 | 0 | NA | NA | 0.713325060039162 | 0.846674939960838 | 0 | 0 | 0.439803919254224 | 0.600196080745776 | 0 | 0 | 0 | 0 | 0 | 0 |
|  | Laos | 2000 | 6 | 4 | 67 | 0 | 0 | 0 | 0 | 0 | 0 | 0 | 0 | 0 | 0 | 4 | 100 | 0.292797824129445 | 1.04720217587056 | 0 | 0 | 0 | 0 | 0 | 0 | 0 | 0 | 0 | 0 |
|  | Laos | 2001 | 9 | 3 | 33 | 0 | 0 | 2 | 22 | 0 | 0 | 0 | 0 | 0 | 0 | 3 | 100 | 0.0220157130831926 | 0.637984286916807 | 0 | 0 | -0.0516166102991454 | 0.491616610299145 | 0 | 0 | 0 | 0 | 0 | 0 |
|  | Laos | 2002 | 21 | 1 | 5 | 0 | 0 | 0 | 0 | 0 | 0 | 0 | 0 | 0 | 0 | 1 | 100 | -0.0410840068085297 | 0.14108400680853 | 0 | 0 | 0 | 0 | 0 | 0 | 0 | 0 | 0 | 0 |
|  | Laos | 2003 | 19 | 3 | 16 | 0 | 0 | 0 | 0 | 0 | 0 | 0 | 0 | 0 | 0 | 3 | 100 | -0.00396316335878855 | 0.323963163358789 | 0 | 0 | 0 | 0 | 0 | 0 | 0 | 0 | 0 | 0 |
|  | Laos | 2004 | 11 | 2 | 18 | 0 | 0 | 1 | 9 | 0 | 0 | 0 | 0 | 0 | 0 | 2 | 100 | -0.0479309476674526 | 0.407930947667453 | 0 | 0 | -0.0798896977201237 | 0.259889697720124 | 0 | 0 | 0 | 0 | 0 | 0 |
|  | Laos | 2007 | 5 | 0 | 0 | 0 | 0 | 0 | 0 | 0 | 0 | 0 | 0 | 0 | 0 | 0 | NA | 0 | 0 | 0 | 0 | 0 | 0 | 0 | 0 | 0 | 0 | 0 | 0 |
|  | Laos | 2008 | 21 | 1 | 5 | 0 | 0 | 1 | 5 | 0 | 0 | 0 | 0 | 0 | 0 | 1 | 100 | -0.0410840068085297 | 0.14108400680853 | 0 | 0 | -0.0410840068085297 | 0.14108400680853 | 0 | 0 | 0 | 0 | 0 | 0 |
|  | Laos | 2009 | 20 | 0 | 0 | 0 | 0 | 0 | 0 | 0 | 0 | 0 | 0 | 0 | 0 | 0 | NA | 0 | 0 | 0 | 0 | 0 | 0 | 0 | 0 | 0 | 0 | 0 | 0 |
|  | Laos | 2010 | 26 | 0 | 0 | 0 | 0 | 0 | 0 | 0 | 0 | 0 | 0 | 0 | 0 | 0 | NA | 0 | 0 | 0 | 0 | 0 | 0 | 0 | 0 | 0 | 0 | 0 | 0 |
|  | Laos | 2018 | 1 | 0 | 0 | 0 | 0 | 0 | 0 | 0 | 0 | 0 | 0 | 0 | 0 | 0 | NA | 0 | 0 | 0 | 0 | 0 | 0 | 0 | 0 | 0 | 0 | 0 | 0 |
|  | Laos | all | 27 | 0 | 0 | 0 | 0 | 0 | 0 | 0 | 0 | 0 | 0 | 0 | 0 | NA | NA | 0 | 0 | 0 | 0 | 0 | 0 | 0 | 0 | 0 | 0 | 0 | 0 |
|  | Lebanon | all | 4 | 0 | 0 | 0 | 0 | 2 | 50 | 1 | 25 | 0 | 0 | 0 | 0 | NA | NA | 0 | 0 | 0 | 0 | 0.01 | 0.99 | -0.174352447854375 | 0.674352447854375 | 0 | 0 | 0 | 0 |
|  | Madagascar | all | 8 | 0 | 0 | 0 | 0 | 0 | 0 | 0 | 0 | 0 | 0 | 0 | 0 | NA | NA | 0 | 0 | 0 | 0 | 0 | 0 | 0 | 0 | 0 | 0 | 0 | 0 |
|  | Malawi | 2004 | 1 | 0 | 0 | 0 | 0 | 0 | 0 | 0 | 0 | 0 | 0 | 0 | 0 | 0 | NA | 0 | 0 | 0 | 0 | 0 | 0 | 0 | 0 | 0 | 0 | 0 | 0 |
|  | Malawi | 2005 | 2 | 0 | 0 | 0 | 0 | 0 | 0 | 0 | 0 | 0 | 0 | 0 | 0 | 0 | NA | 0 | 0 | 0 | 0 | 0 | 0 | 0 | 0 | 0 | 0 | 0 | 0 |
|  | Malawi | 2006 | 3 | 0 | 0 | 0 | 0 | 0 | 0 | 0 | 0 | 0 | 0 | 0 | 0 | 0 | NA | 0 | 0 | 0 | 0 | 0 | 0 | 0 | 0 | 0 | 0 | 0 | 0 |
|  | Malawi | 2007 | 1 | 0 | 0 | 0 | 0 | 0 | 0 | 0 | 0 | 0 | 0 | 0 | 0 | 0 | NA | 0 | 0 | 0 | 0 | 0 | 0 | 0 | 0 | 0 | 0 | 0 | 0 |
|  | Malawi | 2008 | 1 | 0 | 0 | 0 | 0 | 0 | 0 | 0 | 0 | 0 | 0 | 0 | 0 | 0 | NA | 0 | 0 | 0 | 0 | 0 | 0 | 0 | 0 | 0 | 0 | 0 | 0 |
|  | Malawi | 2009 | 3 | 0 | 0 | 0 | 0 | 1 | 33 | 0 | 0 | 0 | 0 | 0 | 0 | 0 | NA | 0 | 0 | 0 | 0 | -0.203444432872781 | 0.863444432872781 | 0 | 0 | 0 | 0 | 0 | 0 |
|  | Malawi | 2010 | 19 | 4 | 21 | 0 | 0 | 3 | 16 | 0 | 0 | 0 | 0 | 0 | 0 | 0 | 0 | 0.026683610461923 | 0.393316389538077 | 0 | 0 | -0.00396316335878855 | 0.323963163358789 | 0 | 0 | 0 | 0 | 0 | 0 |
|  | Malawi | 2011 | 48 | 34 | 71 | 0 | 0 | 3 | 6 | 0 | 0 | 0 | 0 | 0 | 0 | 0 | 0 | 0.581412814200435 | 0.838587185799565 | 0 | 0 | -0.00847958181093106 | 0.128479581810931 | 0 | 0 | 0 | 0 | 0 | 0 |
|  | Malawi | 2012 | 26 | 25 | 96 | 0 | 0 | 0 | 0 | 0 | 0 | 0 | 0 | 0 | 0 | 0 | 0 | 0.886079302909454 | 1.03392069709055 | 0 | 0 | 0 | 0 | 0 | 0 | 0 | 0 | 0 | 0 |
|  | Malawi | 2013 | 8 | 8 | 100 | 0 | 0 | 0 | 0 | 0 | 0 | 0 | 0 | 0 | 0 | 0 | 0 | 1 | 1 | 0 | 0 | 0 | 0 | 0 | 0 | 0 | 0 | 0 | 0 |
|  | Malawi | 2015 | 122 | 122 | 100 | 0 | 0 | 3 | 2 | 0 | 0 | 0 | 0 | 0 | 0 | 0 | 0 | 1 | 1 | 0 | 0 | -0.00748213276874598 | 0.047482132768746 | 0 | 0 | 0 | 0 | 0 | 0 |
|  | Malawi | 2016 | 161 | 157 | 98 | 0 | 0 | 0 | 0 | 0 | 0 | 0 | 0 | 0 | 0 | 0 | 0 | 0.955956533703432 | 1.00404346629657 | 0 | 0 | 0 | 0 | 0 | 0 | 0 | 0 | 0 | 0 |
|  | Malawi | 2017 | 31 | 31 | 100 | 0 | 0 | 0 | 0 | 0 | 0 | 0 | 0 | 0 | 0 | 0 | 0 | 1 | 1 | 0 | 0 | 0 | 0 | 0 | 0 | 0 | 0 | 0 | 0 |
|  | Malawi | 2018 | 42 | 40 | 95 | 0 | 0 | 3 | 7 | 0 | 0 | 0 | 0 | 0 | 0 | 0 | 0 | 0.885593881128047 | 1.01440611887195 | 0 | 0 | -0.00788880963698615 | 0.147888809636986 | 0 | 0 | 0 | 0 | 0 | 0 |
|  | Malawi | 2019 | 101 | 99 | 98 | 0 | 0 | 9 | 9 | 0 | 0 | 0 | 0 | 0 | 0 | 0 | 0 | 0.95282893847802 | 1.00717106152198 | 0 | 0 | 0.0344365531579719 | 0.145563446842028 | 0 | 0 | 0 | 0 | 0 | 0 |
|  | Malawi | all | 558 | 520 | 93 | 0 | 0 | 21 | 3.8 | 0 | 0 | 0 | 0 | 0 | 0 | NA | NA | 0.909097499471973 | 0.950902500528027 | 0 | 0 | 0.0222092934369382 | 0.0537907065630618 | 0 | 0 | 0 | 0 | 0 | 0 |
|  | Malaysia | all | 3 | 0 | 0 | 0 | 0 | 2 | 67 | 0 | 0 | 0 | 0 | 0 | 0 | NA | NA | 0 | 0 | 0 | 0 | 0.136555567127219 | 1.20344443287278 | 0 | 0 | 0 | 0 | 0 | 0 |
|  | Mali | all | 1 | 0 | 0 | 0 | 0 | 0 | 0 | 0 | 0 | 0 | 0 | 0 | 0 | NA | NA | 0 | 0 | 0 | 0 | 0 | 0 | 0 | 0 | 0 | 0 | 0 | 0 |
|  | Marshall Islands | all | 1 | 0 | 0 | 0 | 0 | 0 | 0 | 0 | 0 | 0 | 0 | 0 | 0 | NA | NA | 0 | 0 | 0 | 0 | 0 | 0 | 0 | 0 | 0 | 0 | 0 | 0 |
|  | Mayotte | all | 3 | 0 | 0 | 0 | 0 | 0 | 0 | 0 | 0 | 0 | 0 | 0 | 0 | NA | NA | 0 | 0 | 0 | 0 | 0 | 0 | 0 | 0 | 0 | 0 | 0 | 0 |
|  | Mexico | all | 58 | 1 | 1.7 | 1 | 1.7 | 14 | 24 | 2 | 3.4 | 1 | 1.7 | 0 | 0 | NA | NA | -0.0165005169589435 | 0.0505005169589435 | -0.0165005169589435 | 0.0505005169589435 | 0.129870304839986 | 0.350129695160014 | -0.0129594598002799 | 0.0809594598002799 | -0.0165005169589435 | 0.0505005169589435 | 0 | 0 |
|  | Morocco | all | 2 | 0 | 0 | 0 | 0 | 1 | 50 | 0 | 0 | 0 | 0 | 0 | 0 | NA | NA | 0 | 0 | 0 | 0 | -0.192964645562817 | 1.19296464556282 | 0 | 0 | 0 | 0 | 0 | 0 |
|  | Mozambique | all | 1 | 1 | 100 | 0 | 0 | 0 | 0 | 0 | 0 | 0 | 0 | 0 | 0 | NA | NA | 1 | 1 | 0 | 0 | 0 | 0 | 0 | 0 | 0 | 0 | 0 | 0 |
|  | Myanmar | all | 49 | 0 | 0 | 0 | 0 | 49 | 100 | 17 | 35 | 0 | 0 | 0 | 0 | NA | NA | 0 | 0 | 0 | 0 | 1 | 1 | 0.216721099546393 | 0.483278900453607 | 0 | 0 | 0 | 0 |
|  | Namibia | all | 1 | 0 | 0 | 0 | 0 | 0 | 0 | 0 | 0 | 0 | 0 | 0 | 0 | NA | NA | 0 | 0 | 0 | 0 | 0 | 0 | 0 | 0 | 0 | 0 | 0 | 0 |
|  | Nepal | 2007 | 1 | 0 | 0 | 0 | 0 | 1 | 100 | 0 | 0 | 0 | 0 | 0 | 0 | 0 | NA | 0 | 0 | 0 | 0 | 1 | 1 | 0 | 0 | 0 | 0 | 0 | 0 |
|  | Nepal | 2008 | 6 | 0 | 0 | 0 | 0 | 3 | 50 | 0 | 0 | 0 | 0 | 0 | 0 | 0 | NA | 0 | 0 | 0 | 0 | 0.0999166753454143 | 0.900083324654586 | 0 | 0 | 0 | 0 | 0 | 0 |
|  | Nepal | 2009 | 17 | 0 | 0 | 0 | 0 | 7 | 41 | 0 | 0 | 0 | 0 | 0 | 0 | 0 | NA | 0 | 0 | 0 | 0 | 0.176045335024953 | 0.643954664975047 | 0 | 0 | 0 | 0 | 0 | 0 |
|  | Nepal | 2010 | 29 | 1 | 3 | 0 | 0 | 18 | 62 | 1 | 3 | 0 | 0 | 0 | 0 | 0 | 0 | -0.03641070493127 | 0.09641070493127 | 0 | 0 | 0.443399605400588 | 0.796600394599412 | -0.03641070493127 | 0.09641070493127 | 0 | 0 | 0 | 0 |
|  | Nepal | 2011 | 69 | 1 | 1 | 0 | 0 | 59 | 86 | 1 | 1 | 0 | 0 | 0 | 0 | 0 | 0 | -0.0181992067443862 | 0.0381992067443862 | 0 | 0 | 0.776936841535611 | 0.943063158464389 | -0.0181992067443862 | 0.0381992067443862 | 0 | 0 | 0 | 0 |
|  | Nepal | 2012 | 36 | 7 | 19 | 0 | 0 | 32 | 89 | 0 | 0 | 0 | 0 | 0 | 0 | 0 | 0 | 0.0607143452611157 | 0.319285654738884 | 0 | 0 | 0.787338571027731 | 0.992661428972269 | 0 | 0 | 0 | 0 | 0 | 0 |
|  | Nepal | 2013 | 66 | 0 | 0 | 0 | 0 | 55 | 83 | 18 | 27 | 0 | 0 | 0 | 0 | 0 | NA | 0 | 0 | 0 | 0 | 0.740087959181347 | 0.919912040818653 | 0.162552320841379 | 0.377447679158621 | 0 | 0 | 0 | 0 |
|  | Nepal | 2014 | 31 | 2 | 6 | 0 | 0 | 27 | 87 | 5 | 16 | 0 | 0 | 0 | 0 | 0 | 0 | -0.0264823589851068 | 0.146482358985107 | 0 | 0 | 0.751988207128744 | 0.988011792871256 | 0.0305252125440849 | 0.289474787455915 | 0 | 0 | 0 | 0 |
|  | Nepal | 2015 | 40 | 4 | 10 | 0 | 0 | 34 | 85 | 6 | 15 | 0 | 0 | 0 | 0 | 0 | 0 | 0.00702903679104964 | 0.19297096320895 | 0 | 0 | 0.739342420051765 | 0.960657579948235 | 0.0393424200517651 | 0.260657579948235 | 0 | 0 | 0 | 0 |
|  | Nepal | 2016 | 127 | 3 | 2 | 0 | 0 | 107 | 84 | 11 | 9 | 0 | 0 | 0 | 0 | 0 | 0 | -0.00641325717088725 | 0.0464132571708873 | 0 | 0 | 0.776648430815163 | 0.903351569184837 | 0.0410811963890304 | 0.13891880361097 | 0 | 0 | 0 | 0 |
|  | Nepal | 2017 | 262 | 1 | 0 | 0 | 0 | 238 | 91 | 42 | 16 | 0 | 0 | 0 | 0 | 0 | 0 | -0.00746662582449534 | 0.00746662582449534 | 0 | 0 | 0.875070028301187 | 0.944929971698813 | 0.115573702555146 | 0.204426297444854 | 0 | 0 | 0 | 0 |
|  | Nepal | 2018 | 512 | 11 | 2 | 0 | 0 | 481 | 94 | 45 | 9 | 0 | 0 | 0 | 0 | 0 | 0 | 0.00744067394439826 | 0.0325593260556017 | 0 | 0 | 0.919341227409895 | 0.960658772590105 | 0.0654746153730403 | 0.11452538462696 | 0 | 0 | 0 | 0 |
|  | Nepal | 2019 | 103 | 6 | 6 | 0 | 0 | 46 | 45 | 1 | 1 | 0 | 0 | 0 | 0 | 0 | 0 | 0.0147663402810653 | 0.105233659718935 | 0 | 0 | 0.353989976061593 | 0.546010023938407 | -0.00893652650974439 | 0.0289365265097444 | 0 | 0 | 0 | 0 |
|  | Nepal | all | 1275 | 36 | 2.8 | 0 | 0 | 1097 | 86 | 130 | 10 | 0 | 0 | 0 | 0 | NA | NA | 0.0189076174925314 | 0.0370923825074686 | 0 | 0 | 0.840975900481942 | 0.879024099518057 | 0.0833901689987059 | 0.116609831001294 | 0 | 0 | 0 | 0 |
|  | Netherlands | all | 1 | 0 | 0 | 0 | 0 | 1 | 100 | 0 | 0 | 0 | 0 | 0 | 0 | NA | NA | 0 | 0 | 0 | 0 | 1 | 1 | 0 | 0 | 0 | 0 | 0 | 0 |
|  | New Zealand | all | 45 | 0 | 0 | 0 | 0 | 4 | 8.9 | 0 | 0 | 0 | 0 | 0 | 0 | NA | NA | 0 | 0 | 0 | 0 | 0.00585057989919512 | 0.172149420100805 | 0 | 0 | 0 | 0 | 0 | 0 |
|  | Niger | all | 1 | 0 | 0 | 0 | 0 | 0 | 0 | 0 | 0 | 0 | 0 | 0 | 0 | NA | NA | 0 | 0 | 0 | 0 | 0 | 0 | 0 | 0 | 0 | 0 | 0 | 0 |
|  | Nigeria | 2002 | 1 | 0 | 0 | 0 | 0 | 0 | 0 | 0 | 0 | 0 | 0 | 0 | 0 | 0 | NA | 0 | 0 | 0 | 0 | 0 | 0 | 0 | 0 | 0 | 0 | 0 | 0 |
|  | Nigeria | 2008 | 1 | 1 | 100 | 0 | 0 | 0 | 0 | 0 | 0 | 0 | 0 | 0 | 0 | 1 | 100 | 1 | 1 | 0 | 0 | 0 | 0 | 0 | 0 | 0 | 0 | 0 | 0 |
|  | Nigeria | 2009 | 18 | 13 | 72 | 0 | 0 | 1 | 6 | 0 | 0 | 0 | 0 | 0 | 0 | 13 | 100 | 0.513079274704231 | 0.926920725295769 | 0 | 0 | -0.0458209788323777 | 0.165820978832378 | 0 | 0 | 0 | 0 | 0 | 0 |
|  | Nigeria | 2010 | 29 | 14 | 48 | 0 | 0 | 1 | 3 | 0 | 0 | 0 | 0 | 0 | 0 | 14 | 100 | 0.298126794246619 | 0.661873205753381 | 0 | 0 | -0.03641070493127 | 0.09641070493127 | 0 | 0 | 0 | 0 | 0 | 0 |
|  | Nigeria | 2011 | 8 | 5 | 62 | 0 | 0 | 0 | 0 | 0 | 0 | 0 | 0 | 0 | 0 | 5 | 100 | 0.284519933528086 | 0.955480066471914 | 0 | 0 | 0 | 0 | 0 | 0 | 0 | 0 | 0 | 0 |
|  | Nigeria | 2012 | 9 | 4 | 44 | 0 | 0 | 0 | 0 | 0 | 0 | 0 | 0 | 0 | 0 | 4 | 100 | 0.115356056600031 | 0.76464394339997 | 0 | 0 | 0 | 0 | 0 | 0 | 0 | 0 | 0 | 0 |
|  | Nigeria | 2013 | 65 | 29 | 45 | 0 | 0 | 5 | 8 | 0 | 0 | 0 | 0 | 0 | 0 | 29 | 100 | 0.329152885325422 | 0.570847114674578 | 0 | 0 | 0.0152191467792021 | 0.144780853220798 | 0 | 0 | 0 | 0 | 0 | 0 |
|  | Nigeria | 2015 | 4 | 1 | 25 | 0 | 0 | 2 | 50 | 0 | 0 | 0 | 0 | 0 | 0 | 1 | 100 | -0.174352447854375 | 0.674352447854375 | 0 | 0 | 0.01 | 0.99 | 0 | 0 | 0 | 0 | 0 | 0 |
|  | Nigeria | 2016 | 5 | 1 | 20 | 0 | 0 | 4 | 80 | 0 | 0 | 0 | 0 | 0 | 0 | 1 | 100 | -0.150615458871967 | 0.550615458871967 | 0 | 0 | 0.449384541128033 | 1.15061545887197 | 0 | 0 | 0 | 0 | 0 | 0 |
|  | Nigeria | 2017 | 21 | 2 | 10 | 0 | 0 | 13 | 62 | 0 | 0 | 0 | 0 | 0 | 0 | 2 | 100 | -0.0255506390384492 | 0.225550639038449 | 0 | 0 | 0.412296507634704 | 0.827703492365296 | 0 | 0 | 0 | 0 | 0 | 0 |
|  | Nigeria | 2018 | 4 | 1 | 25 | 0 | 0 | 3 | 75 | 0 | 0 | 0 | 0 | 0 | 0 | 1 | 100 | -0.174352447854375 | 0.674352447854375 | 0 | 0 | 0.325647552145625 | 1.17435244785437 | 0 | 0 | 0 | 0 | 0 | 0 |
|  | Nigeria | 2019 | 5 | 1 | 20 | 0 | 0 | 4 | 80 | 0 | 0 | 0 | 0 | 0 | 0 | 1 | 100 | -0.150615458871967 | 0.550615458871967 | 0 | 0 | 0.449384541128033 | 1.15061545887197 | 0 | 0 | 0 | 0 | 0 | 0 |
|  | Nigeria | all | 150 | 58 | 39 | 0 | 0 | 32 | 21 | 0 | 0 | 0 | 0 | 0 | 0 | NA | NA | 0.312065977381624 | 0.467934022618376 | 0 | 0 | 0.144440569003441 | 0.275559430996559 | 0 | 0 | 0 | 0 | 0 | 0 |
|  | Pakistan | 2003 | 34 | 8 | 24 | 0 | 0 | 13 | 38 | 0 | 0 | 0 | 0 | 0 | 0 | 8 | 100 | 0.0974164697522764 | 0.382583530247724 | 0 | 0 | 0.216650044946445 | 0.543349955053555 | 0 | 0 | 0 | 0 | 0 | 0 |
|  | Pakistan | 2006 | 2 | 0 | 0 | 0 | 0 | 1 | 50 | 0 | 0 | 0 | 0 | 0 | 0 | 0 | NA | 0 | 0 | 0 | 0 | -0.192964645562817 | 1.19296464556282 | 0 | 0 | 0 | 0 | 0 | 0 |
|  | Pakistan | 2007 | 2 | 1 | 50 | 0 | 0 | 1 | 50 | 0 | 0 | 0 | 0 | 0 | 0 | 0 | 0 | -0.192964645562817 | 1.19296464556282 | 0 | 0 | -0.192964645562817 | 1.19296464556282 | 0 | 0 | 0 | 0 | 0 | 0 |
|  | Pakistan | 2008 | 1 | 0 | 0 | 0 | 0 | 1 | 100 | 0 | 0 | 0 | 0 | 0 | 0 | 0 | NA | 0 | 0 | 0 | 0 | 1 | 1 | 0 | 0 | 0 | 0 | 0 | 0 |
|  | Pakistan | 2009 | 2 | 1 | 50 | 0 | 0 | 1 | 50 | 0 | 0 | 0 | 0 | 0 | 0 | 0 | 0 | -0.192964645562817 | 1.19296464556282 | 0 | 0 | -0.192964645562817 | 1.19296464556282 | 0 | 0 | 0 | 0 | 0 | 0 |
|  | Pakistan | 2010 | 3 | 3 | 100 | 0 | 0 | 3 | 100 | 0 | 0 | 0 | 0 | 0 | 0 | 0 | 0 | 1 | 1 | 0 | 0 | 1 | 1 | 0 | 0 | 0 | 0 | 0 | 0 |
|  | Pakistan | 2011 | 9 | 1 | 11 | 0 | 0 | 7 | 78 | 0 | 0 | 0 | 0 | 0 | 0 | 1 | 100 | -0.0953228579445382 | 0.315322857944538 | 0 | 0 | 0.508383389700855 | 1.05161661029915 | 0 | 0 | 0 | 0 | 0 | 0 |
|  | Pakistan | 2012 | 4 | 1 | 25 | 0 | 0 | 4 | 100 | 0 | 0 | 0 | 0 | 0 | 0 | 0 | 0 | -0.174352447854375 | 0.674352447854375 | 0 | 0 | 1 | 1 | 0 | 0 | 0 | 0 | 0 | 0 |
|  | Pakistan | 2014 | 34 | 21 | 62 | 0 | 0 | 34 | 100 | 0 | 0 | 0 | 0 | 0 | 0 | 0 | 0 | 0.456650044946445 | 0.783349955053555 | 0 | 0 | 1 | 1 | 0 | 0 | 0 | 0 | 0 | 0 |
|  | Pakistan | 2015 | 48 | 20 | 42 | 0 | 0 | 47 | 98 | 4 | 8 | 0 | 0 | 0 | 0 | 0 | 0 | 0.280527614574439 | 0.559472385425561 | 0 | 0 | 0.939594252620496 | 1.0204057473795 | 0.00181011953323239 | 0.158189880466768 | 0 | 0 | 0 | 0 |
|  | Pakistan | 2016 | 96 | 51 | 53 | 2 | 2 | 93 | 97 | 6 | 6 | 2 | 2 | 0 | 0 | 0 | 0 | 0.430174713170334 | 0.629825286829666 | -0.00857117797095787 | 0.0485711779709579 | 0.935194223794151 | 1.00480577620585 | 0.0115776233286717 | 0.108422376671328 | -0.00857117797095787 | 0.0485711779709579 | 0 | 0 |
|  | Pakistan | 2017 | 337 | 200 | 59 | 57 | 17 | 325 | 96 | 69 | 20 | 57 | 17 | 2 | 1 | 0 | 0 | 0.53755712390237 | 0.64244287609763 | 0.129975338877627 | 0.210024661122373 | 0.940214659736276 | 0.979785340263724 | 0.156917275234716 | 0.243082724765284 | 0.129975338877627 | 0.210024661122373 | 0.00179934349305062 | 0.0182006565069494 |
|  | Pakistan | 2018 | 678 | 542 | 80 | 390 | 58 | 673 | 99 | 397 | 59 | 409 | 60 | 0 | 0 | 2 | 0 | 0.769857405717353 | 0.830142594282647 | 0.54279167859509 | 0.61720832140491 | 0.98355973015038 | 0.99644026984962 | 0.552918273714256 | 0.627081726285744 | 0.563174442988659 | 0.636825557011341 | 0 | 0 |
|  | Pakistan | 2019 | 244 | 139 | 57 | 69 | 28 | 241 | 99 | 87 | 36 | 71 | 29 | 0 | 0 | 2 | 1 | 0.507874001549289 | 0.632125998450711 | 0.223491353968839 | 0.336508646031162 | 0.976172601459221 | 1.00382739854078 | 0.299899126455474 | 0.420100873544526 | 0.233006732315863 | 0.346993267684137 | 0 | 0 |
|  | Pakistan | 2020 | 31 | 27 | 87 | 23 | 74 | 31 | 100 | 24 | 77 | 26 | 84 | 0 | 0 | 0 | 0 | 0.751988207128744 | 0.988011792871256 | 0.585963925604752 | 0.894036074395248 | 1 | 1 | 0.622813434492276 | 0.917186565507724 | 0.710525212544085 | 0.969474787455915 | 0 | 0 |
|  | Pakistan | all | 1484 | 1005 | 68 | 541 | 36 | 1458 | 98 | 587 | 40 | 565 | 38 | 2 | 0.13 | NA | NA | 0.65621210529161 | 0.70378789470839 | 0.335511640128309 | 0.384488359871691 | 0.973324702037087 | 0.986675297962913 | 0.375121711941213 | 0.424878288058787 | 0.355294866812839 | 0.404705133187162 | -0.000566570163929243 | 0.00316657016392924 |
|  | Panama | all | 1 | 1 | 100 | 0 | 0 | 1 | 100 | 0 | 0 | 0 | 0 | 0 | 0 | NA | NA | 1 | 1 | 0 | 0 | 1 | 1 | 0 | 0 | 0 | 0 | 0 | 0 |
|  | Papua New Guinea | all | 5 | 0 | 0 | 0 | 0 | 0 | 0 | 0 | 0 | 0 | 0 | 0 | 0 | NA | NA | 0 | 0 | 0 | 0 | 0 | 0 | 0 | 0 | 0 | 0 | 0 | 0 |
|  | Peru | all | 3 | 0 | 0 | 0 | 0 | 2 | 67 | 0 | 0 | 0 | 0 | 0 | 0 | NA | NA | 0 | 0 | 0 | 0 | 0.136555567127219 | 1.20344443287278 | 0 | 0 | 0 | 0 | 0 | 0 |
|  | Philippines | 2011 | 2 | 0 | 0 | 0 | 0 | 0 | 0 | 0 | 0 | 0 | 0 | 0 | 0 | 0 | NA | 0 | 0 | 0 | 0 | 0 | 0 | 0 | 0 | 0 | 0 | 0 | 0 |
|  | Philippines | 2012 | 1 | 0 | 0 | 0 | 0 | 0 | 0 | 0 | 0 | 0 | 0 | 0 | 0 | 0 | NA | 0 | 0 | 0 | 0 | 0 | 0 | 0 | 0 | 0 | 0 | 0 | 0 |
|  | Philippines | 2013 | 76 | 0 | 0 | 0 | 0 | 2 | 3 | 0 | 0 | 1 | 1 | 0 | 0 | 0 | NA | 0 | 0 | 0 | 0 | -0.00598873161188808 | 0.0659887316118881 | 0 | 0 | -0.0156192442748107 | 0.0356192442748107 | 0 | 0 |
|  | Philippines | 2014 | 75 | 0 | 0 | 0 | 0 | 3 | 4 | 0 | 0 | 0 | 0 | 0 | 0 | 0 | NA | 0 | 0 | 0 | 0 | -0.00434973731602026 | 0.0843497373160203 | 0 | 0 | 0 | 0 | 0 | 0 |
|  | Philippines | 2015 | 12 | 0 | 0 | 0 | 0 | 3 | 25 | 0 | 0 | 0 | 0 | 0 | 0 | 0 | NA | 0 | 0 | 0 | 0 | 0.005 | 0.495 | 0 | 0 | 0 | 0 | 0 | 0 |
|  | Philippines | 2016 | 11 | 0 | 0 | 0 | 0 | 6 | 55 | 0 | 0 | 0 | 0 | 0 | 0 | 0 | NA | 0 | 0 | 0 | 0 | 0.255742411866227 | 0.844257588133773 | 0 | 0 | 0 | 0 | 0 | 0 |
|  | Philippines | 2017 | 24 | 0 | 0 | 0 | 0 | 12 | 50 | 0 | 0 | 0 | 0 | 0 | 0 | 0 | NA | 0 | 0 | 0 | 0 | 0.299958337672707 | 0.700041662327293 | 0 | 0 | 0 | 0 | 0 | 0 |
|  | Philippines | 2018 | 1 | 0 | 0 | 0 | 0 | 0 | 0 | 0 | 0 | 0 | 0 | 0 | 0 | 0 | NA | 0 | 0 | 0 | 0 | 0 | 0 | 0 | 0 | 0 | 0 | 0 | 0 |
|  | Philippines | 2019 | 4 | 0 | 0 | 0 | 0 | 1 | 25 | 1 | 25 | 0 | 0 | 0 | 0 | 0 | NA | 0 | 0 | 0 | 0 | -0.174352447854375 | 0.674352447854375 | -0.174352447854375 | 0.674352447854375 | 0 | 0 | 0 | 0 |
|  | Philippines | all | 206 | 0 | 0 | 0 | 0 | 27 | 13 | 1 | 0.49 | 1 | 0.49 | 0 | 0 | NA | NA | 0 | 0 | 0 | 0 | 0.0839145706002237 | 0.176085429399776 | -0.00459144141257714 | 0.0143914414125771 | -0.00459144141257714 | 0.0143914414125771 | 0 | 0 |
|  | Portugal | all | 1 | 0 | 0 | 0 | 0 | 0 | 0 | 0 | 0 | 0 | 0 | 0 | 0 | NA | NA | 0 | 0 | 0 | 0 | 0 | 0 | 0 | 0 | 0 | 0 | 0 | 0 |
|  | Qatar | all | 1 | 1 | 100 | 0 | 0 | 1 | 100 | 0 | 0 | 0 | 0 | 0 | 0 | NA | NA | 1 | 1 | 0 | 0 | 1 | 1 | 0 | 0 | 0 | 0 | 0 | 0 |
|  | Rwanda | all | 27 | 15 | 56 | 0 | 0 | 23 | 85 | 0 | 0 | 0 | 0 | 0 | 0 | NA | NA | 0.372566731887246 | 0.747433268112754 | 0 | 0 | 0.71600014179984 | 0.98399985820016 | 0 | 0 | 0 | 0 | 0 | 0 |
|  | Samoa | 2002 | 2 | 0 | 0 | 0 | 0 | 0 | 0 | 0 | 0 | 0 | 0 | 0 | 0 | 0 | NA | 0 | 0 | 0 | 0 | 0 | 0 | 0 | 0 | 0 | 0 | 0 | 0 |
|  | Samoa | 2004 | 7 | 0 | 0 | 0 | 0 | 0 | 0 | 0 | 0 | 0 | 0 | 0 | 0 | 0 | NA | 0 | 0 | 0 | 0 | 0 | 0 | 0 | 0 | 0 | 0 | 0 | 0 |
|  | Samoa | 2005 | 1 | 0 | 0 | 0 | 0 | 0 | 0 | 0 | 0 | 0 | 0 | 0 | 0 | 0 | NA | 0 | 0 | 0 | 0 | 0 | 0 | 0 | 0 | 0 | 0 | 0 | 0 |
|  | Samoa | 2006 | 1 | 0 | 0 | 0 | 0 | 0 | 0 | 0 | 0 | 0 | 0 | 0 | 0 | 0 | NA | 0 | 0 | 0 | 0 | 0 | 0 | 0 | 0 | 0 | 0 | 0 | 0 |
|  | Samoa | 2007 | 4 | 0 | 0 | 0 | 0 | 1 | 25 | 0 | 0 | 0 | 0 | 1 | 25 | 0 | NA | 0 | 0 | 0 | 0 | -0.174352447854375 | 0.674352447854375 | 0 | 0 | 0 | 0 | -0.174352447854375 | 0.674352447854375 |
|  | Samoa | 2008 | 12 | 0 | 0 | 0 | 0 | 0 | 0 | 0 | 0 | 0 | 0 | 0 | 0 | 0 | NA | 0 | 0 | 0 | 0 | 0 | 0 | 0 | 0 | 0 | 0 | 0 | 0 |
|  | Samoa | 2009 | 9 | 0 | 0 | 0 | 0 | 0 | 0 | 0 | 0 | 0 | 0 | 0 | 0 | 0 | NA | 0 | 0 | 0 | 0 | 0 | 0 | 0 | 0 | 0 | 0 | 0 | 0 |
|  | Samoa | 2010 | 7 | 0 | 0 | 0 | 0 | 0 | 0 | 0 | 0 | 0 | 0 | 0 | 0 | 0 | NA | 0 | 0 | 0 | 0 | 0 | 0 | 0 | 0 | 0 | 0 | 0 | 0 |
|  | Samoa | 2011 | 9 | 0 | 0 | 0 | 0 | 0 | 0 | 0 | 0 | 0 | 0 | 0 | 0 | 0 | NA | 0 | 0 | 0 | 0 | 0 | 0 | 0 | 0 | 0 | 0 | 0 | 0 |
|  | Samoa | 2012 | 58 | 0 | 0 | 0 | 0 | 9 | 16 | 0 | 0 | 0 | 0 | 0 | 0 | 0 | NA | 0 | 0 | 0 | 0 | 0.0668177107498934 | 0.253182289250107 | 0 | 0 | 0 | 0 | 0 | 0 |
|  | Samoa | 2014 | 2 | 0 | 0 | 0 | 0 | 0 | 0 | 0 | 0 | 0 | 0 | 0 | 0 | 0 | NA | 0 | 0 | 0 | 0 | 0 | 0 | 0 | 0 | 0 | 0 | 0 | 0 |
|  | Samoa | 2015 | 1 | 0 | 0 | 0 | 0 | 0 | 0 | 0 | 0 | 0 | 0 | 0 | 0 | 0 | NA | 0 | 0 | 0 | 0 | 0 | 0 | 0 | 0 | 0 | 0 | 0 | 0 |
|  | Samoa | 2016 | 1 | 0 | 0 | 0 | 0 | 0 | 0 | 0 | 0 | 0 | 0 | 0 | 0 | 0 | NA | 0 | 0 | 0 | 0 | 0 | 0 | 0 | 0 | 0 | 0 | 0 | 0 |
|  | Samoa | 2017 | 7 | 0 | 0 | 0 | 0 | 0 | 0 | 0 | 0 | 0 | 0 | 0 | 0 | 0 | NA | 0 | 0 | 0 | 0 | 0 | 0 | 0 | 0 | 0 | 0 | 0 | 0 |
|  | Samoa | 2018 | 77 | 0 | 0 | 0 | 0 | 0 | 0 | 0 | 0 | 0 | 0 | 0 | 0 | 0 | NA | 0 | 0 | 0 | 0 | 0 | 0 | 0 | 0 | 0 | 0 | 0 | 0 |
|  | Samoa | 2019 | 81 | 0 | 0 | 0 | 0 | 3 | 4 | 0 | 0 | 0 | 0 | 0 | 0 | 0 | NA | 0 | 0 | 0 | 0 | -0.0011278940191023 | 0.0811278940191023 | 0 | 0 | 0 | 0 | 0 | 0 |
|  | Samoa | 2020 | 16 | 0 | 0 | 0 | 0 | 1 | 6 | 0 | 0 | 0 | 0 | 0 | 0 | 0 | NA | 0 | 0 | 0 | 0 | -0.0586101149776021 | 0.178610114977602 | 0 | 0 | 0 | 0 | 0 | 0 |
|  | Samoa | all | 259 | 0 | 0 | 0 | 0 | 13 | 5 | 0 | 0 | 0 | 0 | 0 | 0 | NA | NA | 0 | 0 | 0 | 0 | 0.0234083274066881 | 0.0765916725933119 | 0 | 0 | 0 | 0 | 0 | 0 |
|  | Saudi Arabia | all | 3 | 1 | 33 | 0 | 0 | 3 | 100 | 0 | 0 | 0 | 0 | 0 | 0 | NA | NA | -0.203444432872781 | 0.863444432872781 | 0 | 0 | 1 | 1 | 0 | 0 | 0 | 0 | 0 | 0 |
|  | Senegal | all | 8 | 0 | 0 | 0 | 0 | 0 | 0 | 0 | 0 | 0 | 0 | 0 | 0 | NA | NA | 0 | 0 | 0 | 0 | 0 | 0 | 0 | 0 | 0 | 0 | 0 | 0 |
|  | Sierra Leone | all | 2 | 0 | 0 | 0 | 0 | 0 | 0 | 0 | 0 | 0 | 0 | 0 | 0 | NA | NA | 0 | 0 | 0 | 0 | 0 | 0 | 0 | 0 | 0 | 0 | 0 | 0 |
|  | Singapore | all | 4 | 1 | 25 | 0 | 0 | 4 | 100 | 1 | 25 | 0 | 0 | 2 | 50 | NA | NA | -0.174352447854375 | 0.674352447854375 | 0 | 0 | 1 | 1 | -0.174352447854375 | 0.674352447854375 | 0 | 0 | 0.01 | 0.99 |
|  | South Africa | 2004 | 3 | 0 | 0 | 0 | 0 | 0 | 0 | 0 | 0 | 0 | 0 | 0 | 0 | 0 | NA | 0 | 0 | 0 | 0 | 0 | 0 | 0 | 0 | 0 | 0 | 0 | 0 |
|  | South Africa | 2005 | 3 | 1 | 33 | 0 | 0 | 0 | 0 | 0 | 0 | 0 | 0 | 0 | 0 | 0 | 0 | -0.203444432872781 | 0.863444432872781 | 0 | 0 | 0 | 0 | 0 | 0 | 0 | 0 | 0 | 0 |
|  | South Africa | 2006 | 3 | 0 | 0 | 0 | 0 | 0 | 0 | 0 | 0 | 0 | 0 | 0 | 0 | 0 | NA | 0 | 0 | 0 | 0 | 0 | 0 | 0 | 0 | 0 | 0 | 0 | 0 |
|  | South Africa | 2007 | 5 | 0 | 0 | 0 | 0 | 0 | 0 | 0 | 0 | 0 | 0 | 0 | 0 | 0 | NA | 0 | 0 | 0 | 0 | 0 | 0 | 0 | 0 | 0 | 0 | 0 | 0 |
|  | South Africa | 2008 | 4 | 0 | 0 | 0 | 0 | 0 | 0 | 0 | 0 | 0 | 0 | 0 | 0 | 0 | NA | 0 | 0 | 0 | 0 | 0 | 0 | 0 | 0 | 0 | 0 | 0 | 0 |
|  | South Africa | 2009 | 9 | 0 | 0 | 0 | 0 | 0 | 0 | 0 | 0 | 0 | 0 | 0 | 0 | 0 | NA | 0 | 0 | 0 | 0 | 0 | 0 | 0 | 0 | 0 | 0 | 0 | 0 |
|  | South Africa | 2010 | 5 | 1 | 20 | 0 | 0 | 1 | 20 | 1 | 20 | 0 | 0 | 0 | 0 | 0 | 0 | -0.150615458871967 | 0.550615458871967 | 0 | 0 | -0.150615458871967 | 0.550615458871967 | -0.150615458871967 | 0.550615458871967 | 0 | 0 | 0 | 0 |
|  | South Africa | 2011 | 3 | 1 | 33 | 0 | 0 | 0 | 0 | 0 | 0 | 0 | 0 | 0 | 0 | 0 | 0 | -0.203444432872781 | 0.863444432872781 | 0 | 0 | 0 | 0 | 0 | 0 | 0 | 0 | 0 | 0 |
|  | South Africa | 2012 | 8 | 1 | 12 | 0 | 0 | 0 | 0 | 0 | 0 | 0 | 0 | 0 | 0 | 0 | 0 | -0.109176514939904 | 0.349176514939904 | 0 | 0 | 0 | 0 | 0 | 0 | 0 | 0 | 0 | 0 |
|  | South Africa | 2016 | 7 | 2 | 29 | 0 | 0 | 3 | 43 | 1 | 14 | 0 | 0 | 0 | 0 | 1 | 50 | -0.0446640106136302 | 0.62466401061363 | 0 | 0 | 0.0633939444035328 | 0.796606055596467 | -0.119229627936314 | 0.399229627936314 | 0 | 0 | 0 | 0 |
|  | South Africa | 2017 | 76 | 51 | 67 | 0 | 0 | 9 | 12 | 1 | 1 | 0 | 0 | 0 | 0 | 0 | 0 | 0.564369149806455 | 0.775630850193545 | 0 | 0 | 0.0473569050439901 | 0.19264309495601 | -0.0156192442748107 | 0.0356192442748107 | 0 | 0 | 0 | 0 |
|  | South Africa | 2018 | 61 | 30 | 49 | 0 | 0 | 14 | 23 | 4 | 7 | 0 | 0 | 0 | 0 | 0 | 0 | 0.364540719339202 | 0.615459280660798 | 0 | 0 | 0.12447040640113 | 0.33552959359887 | 0.0078803868721335 | 0.132119613127867 | 0 | 0 | 0 | 0 |
|  | South Africa | 2019 | 81 | 60 | 74 | 0 | 0 | 19 | 23 | 0 | 0 | 0 | 0 | 0 | 0 | 0 | 0 | 0.644563651235595 | 0.835436348764405 | 0 | 0 | 0.13772135936518 | 0.32227864063482 | 0 | 0 | 0 | 0 | 0 | 0 |
|  | South Africa | 2020 | 44 | 29 | 66 | 0 | 0 | 12 | 27 | 3 | 7 | 0 | 0 | 0 | 0 | 0 | 0 | 0.519937560880902 | 0.800062439119098 | 0 | 0 | 0.13840400600755 | 0.40159599399245 | -0.00447827551485343 | 0.144478275514853 | 0 | 0 | 0 | 0 |
|  | South Africa | all | 285 | 175 | 61 | 0 | 0 | 58 | 20 | 10 | 3.5 | 0 | 0 | 0 | 0 | NA | NA | 0.553479740354254 | 0.666520259645746 | 0 | 0 | 0.153257094236964 | 0.246742905763036 | 0.013637349848651 | 0.056362650151349 | 0 | 0 | 0 | 0 |
|  | Spain | all | 2 | 0 | 0 | 0 | 0 | 0 | 0 | 0 | 0 | 0 | 0 | 0 | 0 | NA | NA | 0 | 0 | 0 | 0 | 0 | 0 | 0 | 0 | 0 | 0 | 0 | 0 |
|  | Sri Lanka | all | 1 | 0 | 0 | 0 | 0 | 1 | 100 | 0 | 0 | 0 | 0 | 0 | 0 | NA | NA | 0 | 0 | 0 | 0 | 1 | 1 | 0 | 0 | 0 | 0 | 0 | 0 |
|  | Sudan | all | 2 | 0 | 0 | 0 | 0 | 0 | 0 | 0 | 0 | 0 | 0 | 0 | 0 | NA | NA | 0 | 0 | 0 | 0 | 0 | 0 | 0 | 0 | 0 | 0 | 0 | 0 |
|  | Suriname | all | 1 | 0 | 0 | 0 | 0 | 0 | 0 | 0 | 0 | 0 | 0 | 0 | 0 | NA | NA | 0 | 0 | 0 | 0 | 0 | 0 | 0 | 0 | 0 | 0 | 0 | 0 |
|  | Switzerland | all | 1 | 0 | 0 | 0 | 0 | 0 | 0 | 0 | 0 | 0 | 0 | 0 | 0 | NA | NA | 0 | 0 | 0 | 0 | 0 | 0 | 0 | 0 | 0 | 0 | 0 | 0 |
|  | Syria | all | 1 | 0 | 0 | 0 | 0 | 1 | 100 | 0 | 0 | 0 | 0 | 0 | 0 | NA | NA | 0 | 0 | 0 | 0 | 1 | 1 | 0 | 0 | 0 | 0 | 0 | 0 |
|  | Tanzania | all | 18 | 8 | 44 | 0 | 0 | 4 | 22 | 0 | 0 | 0 | 0 | 0 | 0 | NA | NA | 0.21044206615074 | 0.66955793384926 | 0 | 0 | 0.0279380529745705 | 0.41206194702543 | 0 | 0 | 0 | 0 | 0 | 0 |
|  | Thailand | all | 5 | 0 | 0 | 0 | 0 | 3 | 60 | 0 | 0 | 0 | 0 | 0 | 0 | NA | NA | 0 | 0 | 0 | 0 | 0.17058551491595 | 1.02941448508405 | 0 | 0 | 0 | 0 | 0 | 0 |
|  | Timor-Leste | all | 1 | 0 | 0 | 0 | 0 | 0 | 0 | 0 | 0 | 0 | 0 | 0 | 0 | NA | NA | 0 | 0 | 0 | 0 | 0 | 0 | 0 | 0 | 0 | 0 | 0 | 0 |
|  | Tonga | all | 3 | 0 | 0 | 0 | 0 | 0 | 0 | 0 | 0 | 0 | 0 | 0 | 0 | NA | NA | 0 | 0 | 0 | 0 | 0 | 0 | 0 | 0 | 0 | 0 | 0 | 0 |
|  | Tunisia | all | 1 | 0 | 0 | 0 | 0 | 1 | 100 | 0 | 0 | 0 | 0 | 0 | 0 | NA | NA | 0 | 0 | 0 | 0 | 1 | 1 | 0 | 0 | 0 | 0 | 0 | 0 |
|  | Turks and Caicos Islands | all | 1 | 0 | 0 | 0 | 0 | 0 | 0 | 0 | 0 | 0 | 0 | 0 | 0 | NA | NA | 0 | 0 | 0 | 0 | 0 | 0 | 0 | 0 | 0 | 0 | 0 | 0 |
|  | Uganda | all | 36 | 8 | 22 | 0 | 0 | 36 | 100 | 0 | 0 | 0 | 0 | 0 | 0 | NA | NA | 0.0841916948504273 | 0.355808305149573 | 0 | 0 | 1 | 1 | 0 | 0 | 0 | 0 | 0 | 0 |
|  | UK | 2008 | 1 | 0 | 0 | 0 | 0 | 1 | 100 | 0 | 0 | 0 | 0 | 0 | 0 | 0 | NA | 0 | 0 | 0 | 0 | 1 | 1 | 0 | 0 | 0 | 0 | 0 | 0 |
|  | UK | 2012 | 28 | 8 | 29 | 0 | 0 | 24 | 86 | 3 | 11 | 1 | 4 | 0 | 0 | 1 | 12 | 0.122667994693185 | 0.457332005306815 | 0 | 0 | 0.730385186031843 | 0.989614813968157 | -0.004564392373896 | 0.224564392373896 | -0.0287386354243376 | 0.108738635424338 | 0 | 0 |
|  | UK | 2014 | 17 | 2 | 12 | 0 | 0 | 15 | 88 | 1 | 6 | 0 | 0 | 0 | 0 | 0 | 0 | -0.0331592802051694 | 0.273159280205169 | 0 | 0 | 0.72684071979483 | 1.03315928020517 | -0.0518517235461677 | 0.171851723546168 | 0 | 0 | 0 | 0 |
|  | UK | 2015 | 22 | 2 | 9 | 0 | 0 | 10 | 45 | 1 | 5 | 0 | 0 | 0 | 0 | 1 | 50 | -0.0301301573116322 | 0.210130157311632 | 0 | 0 | 0.241928464015011 | 0.658071535984989 | -0.0370425684090933 | 0.137042568409093 | 0 | 0 | 0 | 0 |
|  | UK | 2016 | 14 | 2 | 14 | 0 | 0 | 9 | 64 | 1 | 7 | 0 | 0 | 0 | 0 | 1 | 50 | -0.0433030277982336 | 0.323303027798234 | 0 | 0 | 0.389001992039777 | 0.890998007960223 | -0.0649073756323204 | 0.20490737563232 | 0 | 0 | 0 | 0 |
|  | UK | 2017 | 8 | 3 | 38 | 0 | 0 | 5 | 62 | 1 | 12 | 0 | 0 | 0 | 0 | 0 | 0 | 0.0445199335280858 | 0.715480066471914 | 0 | 0 | 0.284519933528086 | 0.955480066471914 | -0.109176514939904 | 0.349176514939904 | 0 | 0 | 0 | 0 |
|  | UK | 2018 | 10 | 3 | 30 | 0 | 0 | 4 | 40 | 0 | 0 | 0 | 0 | 0 | 0 | 0 | 0 | 0.015969015774687 | 0.584030984225313 | 0 | 0 | 0.0963581056573385 | 0.703641894342661 | 0 | 0 | 0 | 0 | 0 | 0 |
|  | UK | 2019 | 6 | 2 | 33 | 0 | 0 | 4 | 67 | 0 | 0 | 0 | 0 | 0 | 0 | 0 | 0 | -0.0472021758705555 | 0.707202175870556 | 0 | 0 | 0.292797824129445 | 1.04720217587056 | 0 | 0 | 0 | 0 | 0 | 0 |
|  | UK | all | 105 | 22 | 21 | 0 | 0 | 71 | 68 | 7 | 6.7 | 1 | 0.95 | 0 | 0 | NA | NA | 0.132156472235549 | 0.287843527764451 | 0 | 0 | 0.590496348098507 | 0.769503651901494 | 0.0192872794351055 | 0.114712720564894 | -0.00907756512371645 | 0.0280775651237165 | 0 | 0 |
|  | United Arab Emirates | all | 4 | 2 | 50 | 0 | 0 | 4 | 100 | 1 | 25 | 0 | 0 | 0 | 0 | NA | NA | 0.01 | 0.99 | 0 | 0 | 1 | 1 | -0.174352447854375 | 0.674352447854375 | 0 | 0 | 0 | 0 |
|  | USA | 2015 | 2 | 0 | 0 | 0 | 0 | 0 | 0 | 0 | 0 | 0 | 0 | 0 | 0 | 0 | NA | 0 | 0 | 0 | 0 | 0 | 0 | 0 | 0 | 0 | 0 | 0 | 0 |
|  | USA | 2016 | 42 | 4 | 10 | 0 | 0 | 16 | 38 | 0 | 0 | 0 | 0 | 0 | 0 | 0 | 0 | 0.0112222917536081 | 0.188777708246392 | 0 | 0 | 0.233131452072371 | 0.526868547927629 | 0 | 0 | 0 | 0 | 0 | 0 |
|  | USA | 2017 | 44 | 3 | 7 | 0 | 0 | 10 | 23 | 1 | 2 | 0 | 0 | 0 | 0 | 0 | 0 | -0.00447827551485343 | 0.144478275514853 | 0 | 0 | 0.106172668145519 | 0.353827331854481 | -0.024036346916349 | 0.064036346916349 | 0 | 0 | 0 | 0 |
|  | USA | 2018 | 22 | 0 | 0 | 0 | 0 | 8 | 36 | 2 | 9 | 0 | 0 | 0 | 0 | 0 | NA | 0 | 0 | 0 | 0 | 0.158983798792214 | 0.561016201207786 | -0.0301301573116322 | 0.210130157311632 | 0 | 0 | 0 | 0 |
|  | USA | 2019 | 30 | 2 | 7 | 1 | 3 | 13 | 43 | 3 | 10 | 1 | 3 | 0 | 0 | 0 | 0 | -0.0192623266723592 | 0.159262326672359 | -0.0342352159096781 | 0.0942352159096781 | 0.25267485399017 | 0.60732514600983 | -0.00735362127101255 | 0.207353621271013 | -0.0342352159096781 | 0.0942352159096781 | 0 | 0 |
|  | USA | all | 140 | 9 | 6.4 | 1 | 0.71 | 47 | 34 | 6 | 4.3 | 1 | 0.71 | 0 | 0 | NA | NA | 0.0233724231586475 | 0.104627576841352 | -0.00684991039397745 | 0.0210499103939774 | 0.261773406056508 | 0.418226593943492 | 0.00945003725784483 | 0.0765499627421552 | -0.00684991039397745 | 0.0210499103939774 | 0 | 0 |
|  | Vietnam | all | 3 | 1 | 33 | 0 | 0 | 1 | 33 | 0 | 0 | 0 | 0 | 0 | 0 | NA | NA | -0.203444432872781 | 0.863444432872781 | 0 | 0 | -0.203444432872781 | 0.863444432872781 | 0 | 0 | 0 | 0 | 0 | 0 |
|  | Zimbabwe | all | 25 | 19 | 76 | 0 | 0 | 12 | 48 | 1 | 4 | 0 | 0 | 0 | 0 | NA | NA | 0.592583413008149 | 0.927416586991851 | 0 | 0 | 0.284156862770226 | 0.675843137229774 | -0.0368159983336805 | 0.11681599833368 | 0 | 0 | 0 | 0 |

 Go

## Footer

© 2023 GitHub, Inc.

### Footer navigation

- Terms
- Privacy
- Security
- Status
- Docs
- Contact GitHub
- Pricing
- API
- Training
- Blog
- About

You can’t perform that action at this time.
